# Properties of Protein Drug Target Classes

**DOI:** 10.1371/journal.pone.0117955

**Published:** 2015-03-30

**Authors:** Simon C. Bull, Andrew J. Doig

**Affiliations:** Manchester Institute of Biotechnology, Faculty of Life Sciences, The University of Manchester, 131 Princess Street, Manchester M1 7DN, United Kigndom; Kyushu University, JAPAN

## Abstract

Accurate identification of drug targets is a crucial part of any drug development program. We mined the human proteome to discover properties of proteins that may be important in determining their suitability for pharmaceutical modulation. Data was gathered concerning each protein’s sequence, post-translational modifications, secondary structure, germline variants, expression profile and drug target status. The data was then analysed to determine features for which the target and non-target proteins had significantly different values. This analysis was repeated for subsets of the proteome consisting of all G-protein coupled receptors, ion channels, kinases and proteases, as well as proteins that are implicated in cancer. Machine learning was used to quantify the proteins in each dataset in terms of their potential to serve as a drug target. This was accomplished by first inducing a random forest that could distinguish between its targets and non-targets, and then using the random forest to quantify the drug target likeness of the non-targets. The properties that can best differentiate targets from non-targets were primarily those that are directly related to a protein’s sequence (e.g. secondary structure). Germline variants, expression levels and interactions between proteins had minimal discriminative power. Overall, the best indicators of drug target likeness were found to be the proteins’ hydrophobicities, *in vivo* half-lives, propensity for being membrane bound and the fraction of non-polar amino acids in their sequences. In terms of predicting potential targets, datasets of proteases, ion channels and cancer proteins were able to induce random forests that were highly capable of distinguishing between targets and non-targets. The non-target proteins predicted to be targets by these random forests comprise the set of the most suitable potential future drug targets, and should therefore be prioritised when building a drug development programme.

## Introduction

The vast majority of the targets of approved drugs are proteins [1,2]. Knowledge of which proteins are the targets of approved drugs enables the division of the human proteome into two classes: approved drug targets and non-targets. A protein is an approved drug target if it is the target of an approved drug, and a non-target otherwise.

In order for a protein to have any potential as a drug target it must be *druggable*. A druggable protein is one that possesses folds that favour interactions with small drug-like molecules, be they endogenous or extraneous, and therefore is one that contains a binding site [1,3]. These binding sites are expected to have certain characteristics that enable high affinity site-specific binding by the drug-like molecule. As with all drug targets, a potential protein drug target must be linked to a disease process.

Currently there is a lack of knowledge about both the number of proteins that modern pharmaceuticals act on and the number of potentially druggable proteins. Drews proposed one of the first counts of the number of human protein targets, and determined that there were only 417 protein drug targets (excluding anti-infectives acting on bacteria, viruses or parasites) [4]. More recent estimates for the number of protein drug targets have included 218 [5]; a consensus number of 324 [6]; 399, reduced to 120 when only approved drug targets are considered [1], and 435 [7]. In terms of potential drug targets, an analysis by Russ and Lampel [8] identified between 2000 and 3000 proteins that are druggable. Using a purely bioinformatics approach, Bakheet and Doig were able to identify 668 proteins that are not currently approved drug targets, but that have target-like properties [9]. These latter estimates lend credence to the belief that, although the estimate of the number of currently targeted proteins is in the hundreds, the number of proteins that are druggable is substantially larger [5].

While knowledge of the number of proteins that may be amenable to pharmaceutical modulation is valuable, it is also useful to consider the families to which these proteins belong. Rask-Anderson *et al*. found that G-protein-coupled receptors (GPCRs) make up 44% of human drug targets, enzymes 29% and transporter proteins 15% [7]; Overington *et al*. found that over 50% of drugs target GPCRs, nuclear receptors or ion channels [6]; Hopkins and Groom found that enzymes comprise 47% of launched targets, while GPCRs account for 30% [1]; and Zheng *et al*. found that enzymes make up 50% of approved targets [10]. One very evident trend in these findings is the prominence of enzymes and GPCRs in the set of approved drug target proteins. Using the estimate of Fredriksson *et al*. that there are approximately 800 GPCRs coded for by the human genome [11], and the knowledge that there are just over 20,000 human proteins [12], we can estimate that roughly 4% of human proteins are GPCRs. The fraction of GPCRs in the set of approved drug targets can therefore be seen to be vastly greater than would be expected if the set’s composition was proportional to that of all the human proteins. Potential reasons for this discrepancy include: the frequency with which proteins from specific families, such as GPCRs and ion channels, can be found to be involved in human diseases, the nature of the diseases that affect developed countries and the potential difficulty of identifying and exploiting other families of proteins.

In this paper, we investigate properties of major types of drug target proteins, in order to identify rules to predict novel future targets. Target classes were selected based on their sizes and importance.

### Target Types Investigated

#### Antineoplastic

Targeted cancer therapies seek to modulate the activity of specific molecular targets that are believed to have a critical role in tumour growth and/or cancer progression. While these targets may be present in non-cancerous cells, they are often overexpressed or altered in cancerous cells, thereby giving targeted therapies increased selectivity and reduced toxicity over conventional cytotoxic treatments [13,14]. By targeting specific proteins, rather than indiscriminately killing proliferating cells, targeted therapies can be used to interfere with specific aspects of cancer progression. For example, the immortalisation of cancer cells could be attacked via the targeting of telomerase, as it is both specific to cancerous cells and necessary for their survival [15]; molecular alterations that deregulate growth can be corrected, as with Imatinib’s targeting of the BCR-ABL protein in chronic myelogenous leukaemia; or the tumour’s blood supply can be cut off by preventing angiogenesis, as done by the drug Bevacizumab’s inhibition of vascular endothelial growth factor A [16]. Due to their importance in modulating growth factors, tyrosine kinases are an especially useful group of targets [17], with drugs such as Imatinib, Gefitinib, Erlotinib and Sunitinib targeting them. Other important targets include growth factors and proteasomes, inhibition of which can potentially slow a tumour’s proliferation by inhibiting growth/angiogenesis or increasing apoptosis, respectively.

#### GPCRs

The prominent role that GPCRs play in many physiological processes means that GPCRs make up a large fraction of the targets of approved drugs [18,19]. One approach to modulating the activity of a GPCR pharmacologically is to develop a drug that competes with the receptor’s endogenous ligand for access to its orthosteric site. However, in order for a drug to effectively modulate a GPCR’s activity in this manner it must out-compete its endogenous ligand, which necessitates that the drug have a high affinity for the specific GPCR and be maintained at a sufficiently high concentration [20]. Alternatively, a drug can modulate the GPCR’s activity allosterically by binding to a location topographically distinct from the endogenous ligand’s binding site. These allosteric modulators can benefit not only from the increased selectivity due to the often less conserved nature of their binding sites, but also from the fact that the endogenous ligand can still bind to the orthosteric site [21,22].

#### Ion Channels

Ion channels are popular targets for pharmacological intervention due to their key roles in human physiology, localisation in the membrane and pattern of distribution throughout the body [23,24]. The drugs that target them alter their permeability by changing the probability that the channel will be in a given state, often by preferentially binding to and stabilising a particular channel conformation [25]. Pharmacological modulation of ion channels is generally achieved by interacting with the channel’s pore or altering its gating [26]. Pore modulators are primarily inhibitors that exert their effect by binding to the pore and physically or electrostatically blocking the flow of ions [26,27], predominantly by occluding the pore or stabilising a closed or inactive state of the channel. Gating modulators bind to the channel and change the kinetics of the gating process [26]. They are therefore allosteric in nature, and can be designed to enhance the normal conductance of a channel, either positively or negatively, or exert their effect independently of the channel’s gating stimulus [28].

#### Kinases

Kinase activity plays a key role in many cellular processes, such as cell cycle progression, apoptosis, differentiation and signal transduction [29]. Eukaryotic protein kinases are related by a homologous catalytic domain of approximately 250–300 amino acids [30] and can be grouped into the serine/threonine and tyrosine kinases, which are responsible for phosphorylating the hydroxyl oxygen of their respective amino acids. Due to the pivotal role of kinases in the regulation of many cellular processes, aberrant kinase activity has been associated with a variety of diseases and the majority of human cancers [31]. Pharmacological interventions targeting kinases have historically been focussed on the inhibition of malfunctioning kinases, and therefore on preventing irregular kinase activity rather than promoting or enhancing normal activity [31]. These inhibitors can be classified based on the state of the kinase they target (active or inactive) and whether they bind to the active site, an allosteric site or both. The majority of kinase inhibitors developed to date compete directly with ATP for its binding pocket [32,33]. Type I inhibitors rely on the availability of a kinase’s active site, and therefore its active state, while type II inhibitors target the inactive form of the kinase, which can display more structural variation as it is not constrained by the need to catalyse the phosphorylation reaction [34,35].

#### Proteases

Eukaryotic proteases can be divided into ones that perform non-covalent (aspartic and metallo proteases) or covalent (cysteine, serine and threonine proteases) catalysis. Commensurate with their biological importance, deficient or abnormal protease function is present in many pathological conditions. Pharmacological modulation of their activity is therefore a potentially important therapeutic option for treating disease, with an estimated 5–10% of all drugs under development targeting proteases [36]. The therapeutic modulation of protease activity is generally achieved using small molecule reversible or irreversible inhibitors, with the most common approach being to develop a drug that mimics the structure of a protease’s substrate and competes with it for the protease’s active site [37]. Although non-competitive inhibition of protease activity is possible in principle, no non-competitive inhibitors have been approved for sale nor reached the advanced stages of development [38].

## Methods

### Cleaning and Collation of Protein Data

#### Protein Accession and Name

The UniProt accessions and name of each human protein were extracted from an XML file containing all reviewed human proteins from UniProt release 2012_05, hereafter referred to as the UniProt XML file. For each protein <entry> element in the file, the accessions were extracted from its <accession> child elements, and the protein’s name from its <name> child element. The first <accession> element encountered in the record for a protein was taken to be the protein’s representative accession. A mapping between non-representative and representative accessions was produced to enable cross referencing with external databases that may use non-representative accessions. Complete lists of proteins in each set are in S1 Supplementary Information.

#### Simple Sequence Properties

Each protein’s sequence was extracted from the <sequence> child element of its <entry> element in the UniProt XML file. Following the extraction of the sequence, its length was determined by counting the number of amino acid residues in it. Information about the presence or absence of a signal peptide was extracted from the <feature> child elements of a protein’s <entry> element in the UniProt XML file. Any protein with a <feature> element where the value of the **type** attribute was "signal peptide" was deemed to contain a signal peptide.

The number of PEST motifs in each protein was calculated using epestfind (http://emboss.bioinformatics.nl/cgi-bin/emboss/epestfind) which returns potential, poor and invalid PEST motifs. Only potential PEST motifs were counted. The number of PEST motifs returned by epestfind was summed to get the total number of PEST motifs for the protein. The program was run with the default parameters.

The number of low complexity regions was calculated using segmasker [39]. The number of low complexity intervals returned by segmasker was summed to get the total number of low complexity regions for a protein. The program was run with the default parameters.

The hydrophobicity of a protein was calculated to be the mean of the hydrophobicity values, as determined by the Kyte and Doolittle index [40], of the amino acids in its sequence. This was calculated by summing the hydrophobicity values of all the amino acids in the sequence, and then dividing by the sequence length.

The isoelectric point of each protein was calculated using the pepstats program (http://emboss.sourceforge.net/apps/cvs/emboss/apps/pepstats.html). The program was run using the-auto parameter.

#### Amino Acid Composition

Following the extraction of the sequence, the number of occurrences of each of the twenty standard amino acids in the sequence was determined. Ambiguous amino acid codes (B, J and Z) were handled by incrementing the occurrence count for their corresponding amino acids (D/N for B, I/L for Q and E/J for Z) by 0.5. From these occurrence counts, the frequency with which each amino acid occurs in the protein’s sequence was determined by dividing the count for the amino acid by the sequence length. Amino acids were also grouped into eight categories: tiny (A, C, G, S and T), small (A, C, D, G, N, P, S, T and V), aliphatic (I, L and V), aromatic (F, H, W and Y), non-polar (A, C, F, G, I, L, M, P, V, W and Y), charged (D, E, H, K and R), positively charged (H, K and R) and negatively charged (D and E). For each protein, the fraction of the amino acids in its sequence that belong to each of the categories was calculated. This was done by summing up the occurrence counts for each of the amino acids in the category, and then dividing by the length of the sequence.

#### Protein Family

Proteins were classified as being a GPCR, ion channel, kinase, protease or other. Protein family membership was determined using multiple UniProt sources. The first source was the <keyword> child elements of each protein’s <entry> element in the UniProt XML file. A protein was determined to be a GPCR if the value of the **id** attribute of a <keyword> element was "KW-0297"; an ion channel if the value was one of "KW-1071", "KW-0851", "KW-0107", "KW-0869", "KW-0407", "KW-0631" or "KW-0894"; a kinase if the value was one of "KW-0418", "KW-0723" or "KW-0829" and a protease if value was one of "KW-0031", "KW-0064", "KW-0121", "KW-0224", "KW-0482", "KW-0645", "KW-0720", "KW-0788" or "KW-0888". A protein was also determined to be a GPCR, kinase or protease if it appeared in the GPCR (http://www.uniprot.org/docs/7tmrlist accessed May 14th 2012), kinase (http://www.uniprot.org/docs/pkinfam accessed May 14^th^ 2012) or protease (http://www.uniprot.org/docs/peptidas accessed May 14th 2012) files respectively.

For the purposes of this work, a cancer protein is one that is implicated in causing cancer or is the target of an antineoplastic drug. Cancer proteins were determined using two sources: the Cancer Gene Census (CGC) [41] and the FDA’s database of approved drugs. The CGC dataset (accessed on June 15th 2012) was parsed in order to determine the NCBI Gene IDs of genes that are causally implicated in cancer. These were then mapped to representative UniProt human protein accessions.

The FDA’s Drugs@FDA database was downloaded (http://www.fda.gov/downloads/Drugs/InformationOnDrugs/UCM054599.zip accessed April 2013), and processed to determine the set of approved antineoplastic drugs. All drugs approved by the FDA through March 2013 were manually evaluated for evidence of being indicated for antineoplastic use. For each drug, the approved indications for it were determined based on the label data stored by the FDA, or using DrugBank [42] and the Therapeutic Target Database (TTD) [43] if no label data was available. Drugs approved for supportive care (e.g. antiemetics and analgesics), adjunct treatment or non-cancerous cellular proliferation (e.g. actinic keratosis) were excluded from the list, while those approved for precancerous conditions (e.g. myelodysplastic syndrome) were included. Once the final set of approved antineoplastic drugs was created, the DrugBank and TTD Drug IDs of the drugs were determined. The targets of these drugs, as recorded by DrugBank and the TTD, were then determined and converted to representative UniProt accessions.

#### Posttranslational Modifications

Information about the glycosylation and phosphorylation sites of a protein was extracted from the <feature> child elements of the protein’s <entry> element in the UniProt XML file. Information about a glycosylation site was extracted from a <feature> element when the value of its **type** attribute was "glycosylation site". The element’s **description** attribute was used to determine whether the glycosylation was *N*-linked or *O*-linked. Information about a phosphorylation site on the protein was extracted from a <feature> element when the value of its **type** attribute was "modified residue". The element’s **description** attribute was used to determine whether a serine, threonine or tyrosine was phosphorylated. For each protein, the number of each of the five types of posttranslational modification site (*O*-glycosylation, *N*-glycosylation, phosphoserine, phosphothreonine and phosphotyrosine) was calculated. The data on phosphorylation sites extracted from UniProt in this manner was also used to calculate the total number of phosphorylation sites, of any type, for each protein.

#### Secondary Structure

NetSurfP [44] was used to predict the fraction of residues in each protein that participate in exposed *α*-helices, buried *α*-helices or *β*-strands. Although accurate secondary structure information could be obtained from crystal structures, this information is unavailable for the majority of proteins.

Information about the number of *α*-helical transmembrane regions of each protein was extracted from the <feature> child elements of the protein’s <entry> element in the UniProt XML file. A helical transmembrane region is recorded in a <feature> element when its **type** attribute is "transmembrane region" and the **description** attribute is present and contains 'Helical' (without quotes) as its first characters.

#### Protein Protein Interactions

The protein protein interaction (PPI) information for a protein was extracted from the <comment> child elements of the protein’s <entry> element when the value of the **type** attribute was "interaction". PPIs recorded in UniProt can be binary or unary, and can record interactions between human and non-human proteins. For each protein, the number of unique human proteins that participate in a binary interaction with the protein was calculated.

#### External Database References

Data concerning the cross-referencing of UniProt accessions and external database identifiers was extracted from Ensembl [45] using an automated BioMart [46] XML query. The NCBI Gene IDs, Ensembl Gene IDs, Ensembl Transcript IDs, Ensembl Peptide IDs and UniGene cluster IDs associated with each representative UniProt human protein accession were extracted using an XML query. Ensembl variant data was from http://www.ensembl.org/info/genome/variation/sources_documentation.html#homo_sapiens, followed by quality control to weed out bad records. (http://www.ensembl.org/info/genome/variation/data_description.html#quality_control).

#### UniGene Expression Clusters

Unigene [47] was used to extract data relating to the expression profile of the human proteome. Individual transcripts in UniGene are grouped into clusters that are believed to come from the same locus. The expression profile of a cluster is then determined by counting the number of expressed sequence tags (ESTs) in it for each of the body sites and developmental stages recorded in UniGene. The external cross-references extracted from UniProt were used to map UniProt accessions to UniGene cluster IDs from UniGene build #232. A protein’s expression in an individual body site or developmental stage was taken to be the sum of the ESTs in that body site or developmental stage across all UniGene clusters cross-referenced with the protein. In addition to the raw expression values, a derived feature was created that records the number of body sites in which the protein is expressed. This feature was calculated for each protein as the number of body sites in which the expression level was not 0.

#### Ensembl

Ensembl was used to extract information about the alternative transcripts, paralogues and germline variants of UniProt proteins. Details are given in S2 Supplementary Information.

#### Protein Drug Targets

The protein drug targets were determined using the TTD version 4.3.02 [43] and DrugBank version 3 [42]. Details on how UniProt accession numbers were obtained are given in S2 Supplementary Information. The final number of proteins determined to be the target of an approved small molecule drug was 1324, of which 1249 were found in DrugBank and 313 in the TTD. 238 of the proteins were common to both sources, while 1011 were unique to DrugBank and 75 unique to the TTD.

### Machine Learning

#### Datasets Generated

The following 105 features were used in the construction of the protein datasets:

- Amino acid composition
  - Twenty amino acid frequencies
  - Eight amino acid category frequencies
- Simple sequence properties
  - Sequence length
  - The number of PEST motifs
  - The number of low complexity regions
  - The hydrophobicity of the protein
  - The isoelectric point
  - The presence of a signal peptide
- Posttranslational modifications
  - The number of *O*- and *N*-glyosylated sites
  - The number of phosphorylated serine, threonine and tyrosine sites
  - The total number of phosphorylated sites of any type
- Secondary structures
  - The number of *α*-helical transmembrane regions
  - The percentage of residues predicted to participate in an exposed *α*-helix
  - The percentage of residues predicted to participate in a buried *α*-helix
  - The percentage of residues predicted to participate in a *β*-strand
- Germline variants
  - The number of 3’ untranslated region, 5’ untranslated region, nonsynonymous coding and synonymous coding variants
- Inter-protein relationships
  - The number of binary PPIs
  - The number of alternative transcripts
  - The number of paralogues
- Expression levels
  - Seven developmental stage expression levels
  - Forty-five body site expression levels
  - Derived feature recording the number of body sites the protein is expressed in

Six categories were created from the annotated human proteins. Within each category the proteins can be considered to be either positive or negative, positive proteins being those proteins that are approved drug targets and negative proteins those that are not. However, not all positive proteins will have been identified as such yet. Therefore, the set of negative proteins will contain both proteins that will never be the target of an approved drug and those that are not currently but will be in the future. The categories were therefore divided into positive and- unlabelled proteins, rather than positive and negative, where the unlabelled proteins contain both negative and nominally mislabelled positive proteins. Each protein in the human proteome was evaluated against a set of criteria to determine which of the categories it belongs in, and then evaluated against a separate criterion for each category to determine whether it is a positive protein in that specific category. The six categories, along with their criteria, can be seen in Table 1.

**Table 1:** Dataset inclusion criteria.

Category Name	Criterion for Inclusion in Category	# Proteins in Class	Criterion for Inclusion in Positive Class	# Positive Proteins
AllTargets	All proteins are included.	20243	The protein must be a target protein.	1324
Cancer	The protein must be a cancer protein.	831	The protein must be the target of an antineoplastic drug.	387
GPCR	The protein must be a GPCR.	827	The protein must be a target protein.	115
IonChannel	The protein must be an ion channel.	320	The protein must be a target protein.	155
Kinase	The protein must be a kinase.	661	The protein must be a target protein.	94
Protease	The protein must be a protease.	531	The protein must be a target protein.	59

The criteria that a protein must meet to be included in each of the dataset categories, along with the criterion that must be met for each dataset in order to be considered a positive protein in it.

#### Random Forest Parameter Optimisation

In order to make an unbiased prediction about an observation, *i*, the classifier used to make the prediction must not have been trained on a dataset that included *i*. This dilemma leads to the concept of internal generalisation, whereby we want to be able to generalise from our dataset, 𝓓, to an observation i∈𝓓 using some subset of observations, 𝓣⊂𝓓, where i∉𝓣. For the majority of classification algorithms the best way to do this would be to train $\left|𝓓\right|$ classifiers. Each classifier, *c_i_*, is trained using the set of observations ${𝓣}_{i}\;=\;𝓓-i$, and is used to predict the class of observation *i*. This is similar to the leave-one-out cross validation approach used to test classifier performance, but is instead being used to form the final prediction of an observation. However, this approach requires training too many classifiers to be feasible even for small datasets. Rather than using cross validation to train a set of classifiers, a single RF, *R*, can be trained using 𝓓 as the training set. Once *R* has been trained, each observation i∈𝓓 is predicted using only those trees in *R* for which it is OOB, thereby giving an unbiased prediction of the class of *i*. The parameters and feature set used to train *R* can therefore be optimised using 𝓓, while still allowing unbiased predictions of the observations in 𝓓 to be made. In this manner RFs can enable a population dataset to be used as both the training set and the set of observations that are to be predicted, without worrying about the final predictions being biased.

Random forests (RFs) rely on two primary parameters to control their growth: *mtry*, the size of the random subset of features evaluated at each node and *numberTrees*, the number of trees in the forest. In order to mitigate the class imbalances in the datasets used here, the weighting given to observations of the unlabelled class was held at 1 while that of the observations in the positive class was varied. A grid search was used to simultaneously optimise the value of the *mtry* parameter and the positive class weighting. For each combination of *mtry* and positive class weighting, 100 RFs were grown with *numberTrees* = 1000. The Out-of-Bag (OOB) predictions from each of the 100 forests were then collated in order to determine the total number of positive proteins predicted correctly (TPs) positive proteins predicted incorrectly (FNs), unlabelled proteins predicted correctly (TNs) and unlabelled proteins predicted incorrectly (FPs). The sensitivity and specificity of the predictions were then calculated, and used to determine the G mean $(\sqrt{Sensitivity*Specificity)}$ for the parameter combination. Once the search was complete, the optimal parameter combination for the dataset was taken to be the one that produced the forests with the greatest G mean. In order to ensure that the variation in the performance of the classifiers was solely dependent on changing *mtry* and the positive class weighting, the same set of 100 random seeds were used to grow the RFs for each parameter combination. The G mean was the primary measure used to evaluate the performance of the RFs, since this places equal importance on correctly predicting observations of both classes. https://github.com/SimonCB765/RandomForest has the code used.

#### Feature Selection

Feature selection was performed using a modified CHC genetic algorithm (CHC-GA) [48]. Details are given in S2 Supplementary Information.

#### Sequence Identity Comparison

In order to determine the optimal sequence identity threshold for generating the non-redundant dataset of each category, nine non-redundant datasets were created from each of the *CancerTarg, GPCR, IonChannel, Kinase* and *Protease* categories. The *AllTargets* category was not tested as the number of proteins in the category makes the process of experimentally determining the optimal threshold prohibitively time consuming. Rather, the final threshold used was determined based on a consensus of the optimal thresholds for the other five categories. Details on the methods used are given in S2 Supplementary Information.

#### Identification of Targets and Their Properties

For each category, the optimal sequence identity threshold was used to generate a non-redundant dataset. Following this, the values for the positive class weighting and *mtry* parameters were optimised. Once the optimal parameter values had been found, feature selection was performed using the CHC-GA algorithm. In order to allow the GA to converge to potentially different feature sets, multiple repetitions of the CHC-GA were performed. These repetitions were repeated with different values for the *numberTrees* parameter, in order to determine the forest size that gave the best performance. The values of *numberTrees* tested were 500, 1000, 1500, 2000, 2500, 3000, 3500, 4000, 4500 and 5000. The optimal feature set, random seed and *numberTrees* value were taken to be those that induced the fittest individual across all CHC-GA repetitions. Once the optimal feature subset/random seed pair was determined, the final predictions for the proteins in the category were generated. This was done by training a RF on the non-redundant dataset using the optimal positive class weighting, *mtry, numberTrees*, feature subset and random seed, and then generating the final predictions using the OOB predictions for the non-redundant proteins and the predictions from the entire forest for the redundant ones.

In addition to forming predictions for the class of each protein in a category, the importance of the features in the category’s dataset was determined using a test of statistical significance and calculating a measure of the size of the effect of the difference between the positive and unlabelled observations. The statistical significance of each feature was determined using a two-tailed Mann-Whitney U test, with significance determined at the 0.05 level and multiple comparisons corrected for using the Bonferroni method. The effect size was calculated by estimating the probability of superiority (PS), also known as the common language effect size [49], defined by PS = U/m*n, where *U* is the U statistic of the positive observations from the Mann-Whitney U test, *m* the number of positive observations in the dataset and *n* the number of unlabelled observations. Here the PS is the fraction of all possible pairs of a positive and unlabelled protein in which the positive observation has a greater value for the feature than the unlabelled observation. The expression levels extracted from UniGene were not tested for significance, as many were zero, but the derived feature recording the number of body sites a protein is expressed in was. Similarly, the proportions of tiny and small amino acids were not tested for significance, as they had very similar distributions.

## Results

### Sequence Identity Comparison

Most algorithms for removing redundancy from a protein dataset will define the distance between two proteins in the dataset to be a function of their sequences. However, when attempting to induce a classifier using the dataset, the proteins are embedded in a space defined by the dataset’s features. The distance between two proteins in this space is therefore determined by the feature vectors that define them and the classification algorithm used, and may be independent of the sequence similarity distance. Therefore, the distance between two proteins during the redundancy removal may be substantially different to the distance between them during the induction of the classifier. If differences in the distance measures cause proteins that are distant in the feature space to be considered too similar by the redundancy removal algorithm, then the removal of one of the too similar proteins may cause information about the distribution of the proteins in the feature space to be lost, to the detriment of the induced classifier’s capabilities.

In order to evaluate the effect that the difference between the two distance measures has on the induction of a classifier, non-redundant datasets were generated using multiple sequence identity thresholds, and then used to induce RFs. As a lower sequence identity threshold causes there to be a greater difference between the original dataset and the non-redundant one generated from it, using a range of thresholds enables classifier capability to be evaluated when the redundancy removal has different levels of influence on the dataset used for training (the non-redundant dataset). In order to compare the capabilities of the induced classifiers, they were used to classify the proteins in the entire dataset from which their non-redundant training dataset was generated. This enables a RF induced using a non-redundant dataset to be evaluated in terms of its capability of generalising to the entire dataset, and therefore allows the loss of information about the distribution of the proteins in the feature space, caused by the redundancy removal, to be assessed.

When classifying the proteins in a non-redundant *Cancer* dataset, the threshold used to generate the dataset made little difference, as evidenced by the fact that the induced RFs all have G means within 0.02 of each other (Table 2). The G means of the classifications of the proteins in the entire *Cancer* dataset show slightly more variation, but as the lowest G mean is no more than 0.03 lower than that achieved by the RF associated with the 100% threshold, the redundancy removal process is unlikely to have led to a substantial loss of the information in the entire *Cancer* dataset. Despite this, the dataset generated using a 20% threshold induced a RF that classified the non-redundant proteins with a greater G mean than it did the entire set of proteins. It is therefore likely that the use of this particular threshold caused the distribution of the proteins in the non-redundant dataset to be different to that of the proteins in the entire dataset. The decision boundary induced using the non-redundant dataset would then fit the proteins in the entire dataset worse than it does those in the non-redundant dataset, thereby leading the RF to overfit the non-redundant dataset to the point where it classifies the entire dataset with a lower G mean.

**Table 2:** Comparison of RFs induced using non-redundant subsets of the *Cancer* dataset.

Threshold	Non-redundant Observations (Pos/Unl)	Non-redundant Dataset G Mean	Entire Dataset				
			TP	FP	TN	FN	G Mean
20%	403 (178/225)	0.84	293	50	394	94	0.82
30%	519 (236/283)	0.83	309	53	391	78	0.84
40%	625 (285/340)	0.83	326	66	378	61	0.85
50%	695 (316/379)	0.84	328	67	377	59	0.85
60%	742 (343/399)	0.84	313	52	392	74	0.85
70%	785 (367/418)	0.84	312	53	391	75	0.84
80%	806 (379/427)	0.84	318	59	385	69	0.84
90%	818 (385/433)	0.85	318	55	389	69	0.85
100%	831 (387/444)	0.85	332	71	373	55	0.85

For each threshold, a non-redundant dataset was generated using Leaf [68] and used to induce a RF. The RF was then used to classify the proteins in both the non-redundant dataset it was trained on and the entire *Cancer* dataset. The TPs/FNs are the number of positive proteins in the entire dataset predicted correctly/incorrectly, and the TNs/FPs are the number of unlabelled proteins predicted correctly/incorrectly.

The threshold used to generate the non-redundant *GPCR* dataset had a substantial effect on the classifications of the proteins in both the non-redundant training set and the entire *GPCR* dataset, with smaller thresholds generally leading to the induction of RFs with lower G means (Table 3). Despite this trend, the largest G mean on the non-redundant datasets is associated with a threshold of 20%. This is likely due to the large fraction of proteins removed at this threshold causing the remaining proteins to be sparsely distributed throughout the feature space, thereby making the decision boundary easier to optimise to fit the distribution of the non-redundant proteins. In addition to this, the 20% and 30% thresholds lead to the induction of RFs that classify the non-redundant proteins with a greater G mean than they do the entire set of proteins. As with the other datasets, this is likely due to the subset of proteins kept by the redundancy removal having a different distribution when compared to the proteins in the entire dataset, thereby leading the RF to overfit the non-redundant dataset to the point where it classifies the proteins in the entire dataset with a lower G mean. Unlike 20% and 30%, the thresholds between 40% and 90% led to the induction of RFs that performed better on the entire *GPCR* dataset than on the non-redundant dataset they were trained on. This is likely due to the redundancy removal predominantly thinning out clusters of proteins in the feature space that belong to a single class, thereby disproportionately removing those proteins that are easier to classify and so reducing the G mean. If this is the case, the distribution of the proteins in the non-redundant dataset will be similar to that of the proteins in the entire dataset. The non-redundant dataset will therefore still contain enough information to correctly classify the vast majority of the removed proteins, and as a result the G mean will be greater when classifying the entire dataset. In addition, this shows that at these thresholds the redundancy removal and classification distance measures are well correlated, causing the redundancy removal to remove proteins that are both similar in terms of sequence identity and their location within the feature space.

**Table 3:** Comparison of RFs induced using non-redundant subsets of the *GPCR* dataset.

Threshold	Non-redundant Observations (Pos/Unl)	Non-redundant Dataset G Mean	Entire Dataset				
			TP	FP	TN	FN	G Mean
20%	57 (14/43)	0.87	80	211	501	35	0.70
30%	150 (39/111)	0.76	94	372	340	21	0.62
40%	276 (66/210)	0.76	93	104	608	22	0.83
50%	409 (86/323)	0.79	90	75	637	25	0.84
60%	556 (102/454)	0.82	93	81	631	22	0.85
70%	665 (111/554)	0.83	93	85	627	22	0.84
80%	735 (113/622)	0.85	98	95	617	17	0.86
90%	779 (114/665)	0.85	99	107	605	16	0.86
100%	827 (115/712)	0.86	100	109	603	15	0.86

For each threshold, a non-redundant dataset was generated using Leaf and used to induce a RF. The RF was then used to classify the proteins in both the non-redundant dataset it was trained on and the entire *GPCR* dataset. The TPs/FNs are the number of positive proteins in the entire dataset predicted correctly/incorrectly, and the TNs/FPs are the number of unlabelled proteins predicted correctly/incorrectly.

The results for the *IonChannel* (Table 4), *Kinase* (Table 5) and *Protease* (Table 6) datasets show the same general trends as the *Cancer* and *GPCR* ones, such as there being less variation in the G means of the non-redundant dataset classifications and larger thresholds leading to the induction of RFs that better classify the entire dataset. All three datasets also exhibit the same trend that once the threshold is below a certain value, 50% in the case of the *IonChannel* dataset and 40% in the case of the *Kinase* and *Protease* datasets, the G mean of the classifications of the entire dataset becomes sizably less than the G mean of the classifications of the non-redundant dataset. Similar to the *Cancer* and *GPCR* datasets, this is likely due to differences in the distribution of the proteins in the non-redundant dataset and those in the entire dataset.

**Table 4:** Comparison of RFs induced using non-redundant subsets of the *IonChannel* dataset.

Threshold	Non-redundant Observations (Pos/Unl)	Non-redundant Dataset G Mean	Entire Dataset				
			TP	FP	TN	FN	G Mean
20%	68 (25/43)	0.82	114	79	86	41	0.62
30%	106 (41/65)	0.81	119	56	109	36	0.71
40%	146 (58/88)	0.80	128	59	106	27	0.73
50%	187 (76/111)	0.82	117	37	128	38	0.77
60%	227 (95/132)	0.81	125	30	135	30	0.81
70%	270 (124/146)	0.82	119	14	151	36	0.84
80%	306 (145/161)	0.85	121	13	152	34	0.85
90%	319 (155/164)	0.85	122	15	150	33	0.85
100%	320 (155/165)	0.85	122	15	150	33	0.85

For each threshold, a non-redundant dataset was generated using Leaf and used to induce a RF. The RF was then used to classify the proteins in both the non-redundant dataset it was trained on and the entire *IonChannel* dataset. The TPs/FNs are the number of positive proteins in the entire dataset predicted correctly/incorrectly, and the TNs/FPs are the number of unlabelled proteins predicted correctly/incorrectly.

**Table 5:** Comparison of RFs induced using non-redundant subsets of the *Kinase* dataset.

Threshold	Non-redundant Observations (Pos/Unl)	Non-redundant Dataset G Mean	Entire Dataset				
			TP	FP	TN	FN	G Mean
20%	102 (18/84)	0.79	51	196	371	43	0.60
30%	198 (26/172)	0.85	49	165	402	45	0.61
40%	332 (49/283)	0.78	75	184	383	19	0.73
50%	432 (67/365)	0.79	72	120	447	22	0.78
60%	497 (77/420)	0.81	77	132	435	17	0.79
70%	569 (83/486)	0.79	72	118	449	22	0.78
80%	625 (88/537)	0.80	72	112	455	22	0.78
90%	650 (94/556)	0.79	69	90	477	25	0.79
100%	661 (94/567)	0.80	72	98	469	22	0.80

For each threshold, a non-redundant dataset was generated using Leaf and used to induce a RF. The RF was then used to classify the proteins in both the non-redundant dataset it was trained on and the entire *Kinase* dataset. The TPs/FNs are the number of positive proteins in the entire dataset predicted correctly/incorrectly, and the TNs/FPs are the number of unlabelled proteins predicted correctly/incorrectly.

**Table 6:** Comparison of RFs induced using non-redundant subsets of the *Protease* dataset.

Threshold	Non-redundant Observations (Pos/Unl)	Non-redundant Dataset G Mean	Entire Dataset				
			TP	FP	TN	FN	G Mean
20%	117 (15/102)	0.90	37	92	380	22	0.71
30%	197 (25/172)	0.84	46	107	365	13	0.78
40%	312 (38/274)	0.86	46	91	381	13	0.79
50%	402 (49/353)	0.83	46	49	423	13	0.84
60%	464 (55/409)	0.85	47	53	419	12	0.84
70%	486 (57/429)	0.85	47	41	431	12	0.85
80%	496 (59/437)	0.86	49	46	426	10	0.87
90%	504 (59/445)	0.86	49	46	426	10	0.87
100%	531 (59/472)	0.87	50	49	423	9	0.87

For each threshold, a non-redundant dataset was generated using Leaf and used to induce a RF. The RF was then used to classify the proteins in both the non-redundant dataset it was trained on and the entire *Protease* dataset. The TPs/FNs are the number of positive proteins in the entire dataset predicted correctly/incorrectly, and the TNs/FPs are the number of unlabelled proteins predicted correctly/incorrectly.

Despite the discussed commonalities in the results for the five datasets, there is a clear difference between the affect that the redundancy removal has on the *Cancer* dataset and the affect that it has on the datasets based on protein family membership. This can be seen most easily through a comparison of the proportion of proteins in the entire dataset that remain following redundancy removal (Table 7). For all thresholds except 90%, the *Cancer* dataset has the greatest proportion of proteins remaining, likely due to the more heterogeneous nature of the proteins in the dataset leading to fewer intra-class similarities between proteins. This lower proportion of proteins removed is also likely responsible for the differences in the threshold at which the induced RFs classify the proteins in the non-redundant dataset with a greater G mean than those in the entire dataset, and for the performance of the RFs induced from non-redundant *Cancer* datasets degrading at a much slower rate than those induced from non-redundant protein family based datasets.

**Table 7:** Fraction of the number of proteins in the entire dataset in each non-redundant dataset.

Threshold	Fraction of Proteins Remaining				
	Cancer	GPCR	IonChannel	Kinase	Protease
20%	0.48	0.07	0.21	0.15	0.22
30%	0.62	0.18	0.33	0.30	0.37
40%	0.75	0.33	0.46	0.50	0.59
50%	0.84	0.49	0.58	0.65	0.76
60%	0.89	0.67	0.71	0.75	0.87
70%	0.94	0.80	0.84	0.86	0.92
80%	0.97	0.89	0.96	0.95	0.93
90%	0.98	0.94	1.00	0.98	0.95

### Target Properties

#### All Proteins

The results from the analysis of the features in the *AllTargets* dataset can be seen in Table 8. Compared to the unlabelled proteins in the dataset, the positive ones are proportionally more non-polar (*PS* = 0.65). Of the individual amino acid proportions, the only non-polar amino acids that occur in a greater proportion in unlabelled proteins are cysteine and proline, and the only polar amino acids that occur in a greater proportion in positive proteins are asparagine, aspartic acid and threonine. However, as the effect sizes for all of these is either small or very small, the differences in the proportions of the individual amino acids can be seen to be largely in line with the difference in proportion of non-polar amino acids. Although the effects of the differences in non-polar and individual amino acids are not large, together they indicate that the positive proteins are consistently more non-polar than the unlabelled ones. This is further demonstrated by the fact that the positive proteins are moderately more hydrophobic (*PS* = 0.67), as would be expected due to their greater proportion of non-polar amino acids and smaller proportion of polar ones. As positive proteins are more likely to contain a transmembrane helix (43% of positive proteins compared to 24% of unlabelled ones), tend to have a greater number of transmembrane *α*-helices (*PS* = 0.60) and have a greater percentage of their residues in buried *α*-helices (*PS* = 0.66), the amino acid composition results are likely due to membrane proteins making up a greater fraction of the set of positive proteins. This is perhaps unsurprising due to the large fraction of membrane proteins (e.g. GPCRs and transport proteins) that are believed to be targeted by approved drugs, and the vital roles in transport and signal transduction that many membrane proteins play. Besides the amino acid proportions, the only other feature with a sizeable effect was the number of *N*-linked glycosylation sites. As *N*-linked glycosylation has been associated with increased protein stability and protection against degradation and denaturation, in addition to ensuring the correct folding of proteins [50,51], the greater number of *N*-linked glycosylation sites likely indicates that positive proteins have a greater half-life *in vivo*. Glycosylation is also very strongly associated with being either a transmembrane or secreted protein.

**Table 8:** Results of the feature analysis for the *AllTargets* dataset.

Feature	P-value	PS	Positive Median	Unlabelled Median	Feature	P-value	PS	Positive Median	Unlabelled Median
Alanine *	3.47 × 10^−04^	0.53	0.07	0.07	Positively Charged *	7.98 × 10^−23^	0.42	0.13	0.14
Arginine *	1.28 × 10^−13^	0.44	0.05	0.06	Sequence Length *	2.13 × 10^−14^	0.56	474	410
Asparagine *	1.33 × 10^−15^	0.57	0.04	0.03	PEST Motifs *	2.66 × 10^−13^	0.45	0	0
Aspartic Acid *	5.90 × 10^−08^	0.54	0.05	0.05	Low Complexity Regions *	1.83 × 10^−08^	0.45	2	2
Cysteine	1.53 × 10^−01^	0.49	0.02	0.02	Hydrophobicity *	3.28 × 10^−93^	0.67	-0.19	-0.38
Glutamic Acid *	3.71 × 10^−19^	0.43	0.06	0.07	Isoelectric Point	1.31 × 10^−01^	0.49	7.31	7.47
Glutamine *	2.57 × 10^−65^	0.36	0.04	0.04	Signal Peptide *	8.10 × 10^−11^	0.53	0	0
Glycine *	2.19 × 10^−10^	0.55	0.07	0.06	*O*-glycosylation Sites *	3.62 × 10^−04^	0.51	0	0
Histidine *	1.35 × 10^−05^	0.46	0.02	0.02	*N*-glycosylation Sites *	1.35 × 10^−64^	0.60	0	0
Isoleucine *	1.10 × 10^−72^	0.65	0.05	0.04	Phosphoserine Sites	7.02 × 10^−01^	0.50	0	0
Leucine *	3.33 × 10^−05^	0.53	0.10	0.10	Phosphothreonine Sites	3.02 × 10^−02^	0.51	0	0
Lysine	1.80 × 10^−01^	0.49	0.05	0.05	Phosphotyrosine Sites *	1.66 × 10^−25^	0.54	0	0
Methionine *	1.31 × 10^−33^	0.60	0.02	0.02	Total Phosphorylation Sites *	1.98 × 10^−04^	0.53	0	0
Phenylalanine *	5.31 × 10^−78^	0.65	0.04	0.04	Transmembrane *α*-helices *	3.16 × 10^−62^	0.60	0	0
Proline *	9.94 × 10^−12^	0.44	0.05	0.06	Exposed *α*-helices *	1.92 × 10^−05^	0.54	0.13	0.12
Serine *	1.37 × 10^−60^	0.37	0.07	0.08	Buried *α*-helices *	2.47 × 10^−89^	0.66	0.22	0.14
Threonine	2.87 × 10^−03^	0.52	0.05	0.05	*β* Strands *	2.40 × 10^−12^	0.56	0.12	0.09
Tryptophan *	4.64 × 10^−24^	0.58	0.01	0.01	3’ Untranslated	7.32 × 10^−01^	0.50	1	1
Tyrosine *	1.61 × 10^−52^	0.63	0.03	0.03	5’ Untranslated	3.41 × 10^−01^	0.51	0	0
Valine *	7.98 × 10^−64^	0.64	0.07	0.06	Nonsynonymous Coding *	6.66 × 10^−16^	0.57	15	11
Aliphatic *	6.09 × 10^−70^	0.65	0.22	0.20	Synonymous Coding *	2.50 × 10^−10^	0.54	0	0
Aromatic *	6.68 × 10^−56^	0.63	0.12	0.10	Binary PPIs *	5.02 × 10^−14^	0.56	1	0
Charged *	1.61 × 10^−131^.61 × 10^−23^	0.42	0.24	0.26	Alternative Transcripts *	2.44 × 10^−18^	0.57	3	2
Negatively Charged *	2.05 × 10^−06^	0.46	0.11	0.11	Paralogues *	5.73 × 10^−07^	0.53	0	0
Non-polar *	1.24 × 10^−72^	0.65	0.56	0.53	Body Sites Expressed In *	5.31 × 10^−12^	0.56	27	26

Shaded features are ones for which the *PS*≥0.5. The amino acid, exposed α-helix, buried α-helix and β strand features are all proportions (e.g. the Alanine feature for a protein is the number of alanine residues in the sequence divided by the sequence length), while all other features are absolute numbers.

Features with significant differences are indicated with *.

Protein interaction and pathway data from KEGG [52], Reactome [53] and STRING [54] were also analysed for the proteins in the dataset. However, the low coverage of these databases made accurate analyses of the proteins in the dataset infeasible. Analysis of the enrichment of Gene Ontology [55] terms between the unlabelled and positive proteins was also investigated using the DAVID [56] functional annotation tool. However, when setting the background to the unlabelled proteins and looking for enriched terms in the positive proteins, no terms were found to be significantly enriched. Identical results were also found when setting the background to the positive proteins and checking for enrichment in the unlabelled proteins or when using the entire proteome as the background set.

#### Cancer Proteins

The results from the analysis of the features in the *Cancer* dataset can be seen in Table 9. Compared to the unlabelled proteins in the dataset, the positive ones have a much greater proportion of non-polar amino acids (*PS* = 0.74). Additionally, the only polar amino acids that occur in a greater proportion in positive proteins are asparagine and threonine (both of which have inconsequential differences in their proportions), while proline is the only non-polar amino acid that occurs in a greater proportion in unlabelled proteins. The positive proteins are also substantially more hydrophobic (*PS* = 0.82), as would be expected due to their greater proportion of non-polar amino acids and smaller proportion of polar ones. As positive proteins are more likely to contain a transmembrane helix than unlabelled ones (55% compared to 11%), tend to have a much greater number of transmembrane helices (*PS* = 0.73) and have a much greater percentage of their residues in buried *α*-helices (*PS* = 0.75), the amino acid composition results are likely due to membrane proteins making up a greater fraction of the set of positive proteins.

**Table 9:** Results of the feature analysis for the *Cancer* dataset.

Feature	P-value	PS	Positive Median	Unlabelled Median	Feature	P-value	PS	Positive Median	Unlabelled Median
Alanine	1.46 × 10^−01^	0.53	0.07	0.07	Positively Charged *	2.63 × 10^−15^	0.34	0.13	0.14
Arginine *	8.88 × 10^−05^	0.42	0.05	0.05	Sequence Length	4.15 × 10^−01^	0.48	505	557
Asparagine	5.26 × 10^−02^	0.54	0.04	0.04	PEST Motifs *	1.06 × 10^−08^	0.40	0	1
Aspartic Acid	1.22 × 10^−02^	0.45	0.05	0.05	Low Complexity Regions *	1.17 × 10^−09^	0.38	2	4
Cysteine *	4.34 × 10^−09^	0.62	0.02	0.02	Hydrophobicity *	1.41 × 10^−57^	0.82	-0.19	-0.57
Glutamic Acid *	3.73 × 10^−12^	0.36	0.06	0.07	Isoelectric Point	1.56 × 10^−01^	0.53	7.04	6.81
Glutamine *	2.43 × 10^−25^	0.29	0.04	0.05	Signal Peptide *	1.11 × 10^−15^	0.61	0	0
Glycine	8.61 × 10^−01^	0.50	0.06	0.06	*O*-glycosylation Sites	7.76 × 10^−01^	0.50	0	0
Histidine	1.80 × 10^−03^	0.44	0.02	0.02	*N*-glycosylation Sites *	2.81 × 10^−38^	0.71	1	0
Isoleucine *	4.15 × 10^−27^	0.72	0.05	0.04	Phosphoserine Sites *	8.17 × 10^−12^	0.37	0	1
Leucine *	4.44 × 10^−16^	0.66	0.10	0.09	Phosphothreonine Sites *	2.13 × 10^−06^	0.42	0	0
Lysine	1.29 × 10^−03^	0.44	0.05	0.06	Phosphotyrosine Sites	3.30 × 10^−03^	0.54	0	0
Methionine *	1.25 × 10^−05^	0.59	0.02	0.02	Total Phosphorylation Sites *	3.31 × 10^−07^	0.40	1	2
Phenylalanine *	1.80 × 10^−42^	0.77	0.04	0.03	Transmembrane *α*-helices *	5.21 × 10^−45^	0.73	1	0
Proline *	2.04 × 10^−13^	0.35	0.05	0.07	Exposed *α*-helices	2.28 × 10^−01^	0.52	0.12	0.11
Serine *	7.94 × 10^−10^	0.38	0.07	0.08	Buried *α*-helices *	1.65 × 10^−35^	0.75	0.22	0.10
Threonine	8.66 × 10^−03^	0.55	0.05	0.05	*β* Strands *	2.67 × 10^−06^	0.59	0.10	0.06
Tryptophan *	6.38 × 10^−27^	0.72	0.02	0.01	3’ Untranslated *	2.55 × 10^−19^	0.32	0	3
Tyrosine *	1.11 × 10^−15^	0.66	0.03	0.02	5’ Untranslated *	1.36 × 10^−16^	0.34	0	2
Valine *	2.70 × 10^−30^	0.73	0.07	0.05	Nonsynonymous Coding *	3.62 × 10^−09^	0.38	14	29
Aliphatic *	5.83 × 10^−43^	0.78	0.22	0.19	Synonymous Coding *	2.04 × 10^−04^	0.44	0	0
Aromatic *	1.85 × 10^−35^	0.75	0.12	0.09	Binary PPIs *	6.83 × 10^−05^	0.42	1	2
Charged *	5.95 × 10^−16^	0.34	0.24	0.26	Alternative Transcripts	1.08 × 10^−02^	0.45	3	4
Negatively Charged *	3.09 × 10^−10^	0.37	0.11	0.12	Paralogues	5.50 × 10^−03^	0.45	0	0
Non-polar *	1.26 × 10^−33^	0.74	0.55	0.51	Body Sites Expressed In *	3.93 × 10^−16^	0.34	24	32

Shaded features are ones for which the *PS*≥0.5. The amino acid, exposed α-helix, buried α-helix and β strand features are all proportions (e.g. the Alanine feature for a protein is the number of alanine residues in the sequence divided by the sequence length), while all other features are absolute numbers.

Features with significant differences are indicated with an *.

As entry to the secretory pathway in humans is controlled by the presence of a signal peptide at the N-terminus of a protein, positive proteins are slightly more likely to be secreted than unlabelled ones due to their increased likelihood of containing a signal peptide (*PS* = 0.61). Additionally, the positive proteins in the *Cancer* dataset are likely to have a longer *in vivo* half-life, due to their greater number of *N*-linked glycosylation sites (*PS* = 0.71), which have been associated with a longer half-life *in vivo*, and smaller number of PEST motifs (*PS* = 0.40), which are associated with proteins with a shorter intracellular half-life [57].

The results also indicate that specific and reliable activity of a cancer protein is likely important in its being targeted by antineoplastic drugs. One example of this is the smaller number of 5’ untranslated (*PS* = 0.34), 3’ untranslated (*PS* = 0.32) and nonsynonymous coding (*PS* = 0.38) variants that are found in the positive proteins. As the untranslated regions of a gene are important for the regulation of mRNA translation and protein expression [58] and nonsynonymous coding variants can lead to alterations in the expression and structure/function of a protein, the activity of a protein with fewer of these variants is likely to be more consistent between individuals.

Further examples of the preference for proteins with reliable activity come from the smaller number of phosphorylation sites (*PS* = 0.40), binary PPIs (*PS* = 0.42) and low complexity regions (*PS* = 0.38) that are found in positive proteins. As protein phosphorylation is frequently altered in cancerous cells, by having fewer phosphorylation sites it is possible that the positive proteins will be less affected by aberrant phosphorylation, thereby ensuring that their activity and its regulation is minimally affected by the cancerous microenvironment. Participating in fewer binary PPIs can also be seen in this light, as a limited set of interactions may make a protein’s activity less susceptible to alterations in the activity or regulation of other proteins. Similarly, it has been shown that low complexity region containing proteins have more binding partners [59], that hub proteins in PPI networks contain significantly more low complexity regions [60,61] and that many known disordered regions in proteins are implicated in signalling and regulation [62]. It therefore seems likely that low complexity regions enable a protein to interact with other proteins more readily, whether in a signalling or regulatory capacity. Having fewer of them may then indicate that a protein is involved in fewer interactions with other proteins, which would in turn imply that the protein’s activity and expression is less amenable to modification by the cancerous microenvironment.

The smaller number of germline variants of all types in positive proteins is possibly a reflection of the predisposition to cancer caused by some germline variants, or may indicate that having fewer viable germline variants means that a protein is less amenable to somatic mutations that leave the protein functional. This would be advantageous for an antineoplastic target, as the cancer microenvironment makes it more likely that genetic mutations will arise in the gene coding for a given protein. If these mutations leave the protein functional, then drugs targeting the protein could have unexpected effects. By targeting proteins that are less susceptible to mutations that leave them viable, the activity of an antineoplastic drug would be more reliable, as the expression and function of the protein itself is more reliable.

The expression of the positive proteins in fewer body sites (*PS* = 0.34) means that the effects of a drug’s modulatory activity can be limited to a more specific range of tissues. Not only can this help to limit undesirable side effects, but also to restrict the activity of the drug to a narrow range of tissues where the cancerous cells originate from. This may be particularly important for antineoplastic drugs, as they can often be more harmful to normal cells than non-antineoplastic medications.

#### GPCRs

The results from the analysis of the features in the *GPCR* dataset can be seen in Table 10. Considering the size and composition of the *GPCR* dataset, when compared to the other datasets investigated, the number of features with meaningful effect sizes is surprisingly large. This was indicative of either substantial differences between the positive and unlabelled proteins or of a large subpopulation of GPCRs (most likely unlabelled ones) that are considerably different to the other proteins in the dataset. A likely contender for this subpopulation is odorant/olfactory GPCRs. While odorant GPCRs are restricted to cells specialised for the detection of external stimuli, e.g. odours and tastes, non-odorant GPCRs are differentially expressed throughout the body, respond to a variety of endogenous ligands and regulate various vital physiological processes [63]. Therefore, non-odorant GPCRs should be more likely to be targeted by drugs. Analysis of the *GPCR* dataset supports this belief, as of the 421 odorant GPCRs in the dataset, none were classified as likely to be a potential drug target or were the target of an approved drug.

**Table 10:** Results of the feature analysis for the *GPCR* dataset.

Feature	P-value	PS	Positive Median	Unlabelled Median	Feature	P-value	PS	Positive Median	Unlabelled Median
Alanine *	4.07 × 10^−08^	0.66	0.08	0.06	Positively Charged *	1.75 × 10^−13^	0.71	0.11	0.10
Arginine *	9.88 × 10^−22^	0.77	0.05	0.04	Sequence Length *	1.97 × 10^−30^	0.81	408	320
Asparagine	1.27 × 10^−03^	0.59	0.04	0.03	PEST Motifs *	2.31 × 10^−07^	0.59	0	0
Aspartic Acid *	5.45 × 10^−08^	0.66	0.03	0.03	Low Complexity Regions *	2.78 × 10^−07^	0.64	2	1
Cysteine	3.63 × 10^−03^	0.42	0.03	0.03	Hydrophobicity *	1.06 × 10^−33^	0.17	0.31	0.68
Glutamic Acid *	6.09 × 10^−14^	0.71	0.03	0.03	Isoelectric Point *	5.97 × 10^−06^	0.63	9.02	8.52
Glutamine	6.78 × 10^−03^	0.58	0.03	0.03	Signal Peptide	1.38 × 10^−03^	0.55	0	0
Glycine *	2.50 × 10^−04^	0.61	0.05	0.05	*O*-glycosylation Sites	1.92 × 10^−02^	0.51	0	0
Histidine *	1.52 × 10^−16^	0.27	0.02	0.03	*N*-glycosylation Sites *	6.47 × 10^−12^	0.68	2	1
Isoleucine *	7.68 × 10^−06^	0.37	0.07	0.08	Phosphoserine Sites *	1.43 × 10^−06^	0.57	0	0
Leucine *	1.86 × 10^−15^	0.28	0.12	0.14	Phosphothreonine Sites *	6.08 × 10^−06^	0.54	0	0
Lysine *	8.30 × 10^−04^	0.60	0.04	0.03	Phosphotyrosine Sites	5.49 × 10^−03^	0.52	0	0
Methionine *	1.17 × 10^−13^	0.29	0.02	0.03	Total Phosphorylation Sites *	7.87 × 10^−08^	0.59	0	0
Phenylalanine *	1.93 × 10^−17^	0.26	0.05	0.07	Transmembrane *α*-helices	9.85 × 10^−01^	0.50	7	7
Proline *	4.39 × 10^−11^	0.69	0.05	0.04	Exposed *α*-helices	3.35 × 10^−01^	0.53	0.09	0.09
Serine	5.60 × 10^−02^	0.44	0.08	0.08	Buried *α*-helices *	6.29 × 10^−19^	0.25	0.47	0.58
Threonine *	7.45 × 10^−04^	0.40	0.06	0.06	*β* Strands *	1.86 × 10^−12^	0.30	0.03	0.04
Tryptophan *	3.41 × 10^−21^	0.76	0.02	0.01	3’ Untranslated	1.81 × 10^−02^	0.53	0	0
Tyrosine *	1.64 × 10^−08^	0.34	0.03	0.04	5’ Untranslated	5.56 × 10^−02^	0.53	0	0
Valine	2.47 × 10^−01^	0.47	0.08	0.08	Nonsynonymous Coding *	3.92 × 10^−16^	0.70	2	0
Aliphatic *	8.59 × 10^−24^	0.22	0.26	0.30	Synonymous Coding	7.95 × 10^−02^	0.52	0	0
Aromatic *	3.23 × 10^−17^	0.26	0.12	0.15	Binary PPIs	6.93 × 10^−03^	0.54	0	0
Charged *	7.68 × 10^−22^	0.77	0.18	0.15	Alternative Transcripts *	6.68 × 10^−18^	0.72	1	0
Negatively Charged *	9.12 × 10^−18^	0.74	0.07	0.05	Paralogues	3.72 × 10^−02^	0.52	0	0
Non-polar *	2.45 × 10^−12^	0.30	0.61	0.65	Body Sites Expressed In *	5.85 × 10^−27^	0.79	12	5

Shaded features are ones for which the *PS*≥0.5. The amino acid, exposed α-helix, buried α-helix and β strand features are all proportions (e.g. the Alanine feature for a protein is the number of alanine residues in the sequence divided by the sequence length), while all other features are absolute numbers.

Features with significant differences are indicated with an *.

In order to evaluate the impact of the odorant GPCRs on the feature analysis, a second dataset, *GPCR_NO*, was constructed from the *GPCR* dataset by removing all odorant GPCRs from it. The results of the analysis of this second dataset can be seen in Table 11. For all features, except the fraction of residues in exposed *α*-helices, the effect size was smaller in the *GPCR_NO* dataset than in the *GPCR* dataset. Additionally, only five features were deemed to have significant differences, compared to thirty-seven features in the *GPCR* dataset.

**Table 11:** Results of the feature analysis for the *GPCR_NO* dataset.

Feature	P-value	PS	Positive Median	Unlabelled Median	Feature	P-value	PS	Positive Median	Unlabelled Median
Alanine	4.75 × 10^−02^	0.56	0.08	0.07	Positively Charged	1.92 × 10^−01^	0.54	0.11	0.11
Arginine	5.63 × 10^−03^	0.59	0.05	0.05	Sequence Length	2.87 × 10^−03^	0.59	408	373
Asparagine	1.77 × 10^−01^	0.54	0.04	0.04	PEST Motifs	2.40 × 10^−01^	0.53	0	0
Aspartic Acid *	7.06 × 10^−04^	0.61	0.03	0.03	Low Complexity Regions	4.71 × 10^−01^	0.48	2	2
Cysteine	4.56 × 10^−01^	0.48	0.03	0.03	Hydrophobicity *	1.84 × 10^−04^	0.38	0.31	0.43
Glutamic Acid	5.66 × 10^−02^	0.56	0.03	0.03	Isoelectric Point	1.81 × 10^−01^	0.54	9.02	8.68
Glutamine	4.25 × 10^−01^	0.47	0.03	0.03	Signal Peptide	5.96 × 10^−01^	0.48	0	0
Glycine	4.89 × 10^−01^	0.52	0.05	0.05	*O*-glycosylation Sites	7.97 × 10^−02^	0.51	0	0
Histidine *	9.56 × 10^−09^	0.32	0.02	0.02	*N*-glycosylation Sites	2.04 × 10^−01^	0.54	2	2
Isoleucine	4.73 × 10^−01^	0.52	0.07	0.06	Phosphoserine Sites	7.94 × 10^−02^	0.53	0	0
Leucine *	1.44 × 10^−04^	0.38	0.12	0.13	Phosphothreonine Sites	5.07 × 10^−03^	0.53	0	0
Lysine	7.18 × 10^−02^	0.56	0.04	0.04	Phosphotyrosine Sites	2.78 × 10^−01^	0.51	0	0
Methionine	4.69 × 10^−01^	0.52	0.02	0.02	Total Phosphorylation Sites	3.51 × 10^−02^	0.55	0	0
Phenylalanine	2.43 × 10^−03^	0.40	0.05	0.06	Transmembrane *α*-helices	9.58 × 10^−01^	0.50	7	7
Proline	1.77 × 10^−03^	0.60	0.05	0.04	Exposed *α*-helices	5.36 × 10^−02^	0.44	0.09	0.10
Serine	1.82 × 10^−01^	0.46	0.08	0.08	Buried *α*-helices	6.55 × 10^−02^	0.44	0.47	0.51
Threonine	6.27 × 10^−01^	0.48	0.06	0.06	*β* Strands	4.84 × 10^−02^	0.44	0.03	0.03
Tryptophan	7.86 × 10^−01^	0.49	0.02	0.02	3’ Untranslated	2.41 × 10^−01^	0.48	0	0
Tyrosine	4.28 × 10^−01^	0.47	0.03	0.03	5’ Untranslated	1.85 × 10^−01^	0.47	0	0
Valine	5.84 × 10^−01^	0.48	0.08	0.08	Nonsynonymous Coding	2.98 × 10^−01^	0.53	2	1
Aliphatic	3.39 × 10^−03^	0.41	0.26	0.27	Synonymous Coding	4.03 × 10^−01^	0.49	0	0
Aromatic *	6.54 × 10^−06^	0.36	0.12	0.14	Binary PPIs	3.70 × 10^−01^	0.48	0	0
Charged	2.63 × 10^−03^	0.60	0.18	0.17	Alternative Transcripts	6.69 × 10^−03^	0.58	1	1
Negatively Charged	1.64 × 10^−03^	0.60	0.07	0.06	Paralogues	7.19 × 10^−01^	0.50	0	0
Non-polar	3.51 × 10^−02^	0.43	0.61	0.63	Body Sites Expressed In	1.73 × 10^−01^	0.54	12	11

Shaded features are ones for which the *PS*≥0.5. The amino acid, exposed α-helix, buried α-helix and β strand features are all proportions (e.g. the Alanine feature for a protein is the number of alanine residues in the sequence divided by the sequence length), while all other features are absolute numbers.

Features with significant differences are indicated with an *.

When compared to the unlabelled proteins, the positive proteins in the *GPCR_NO* dataset have a slightly smaller proportion of non-polar amino acids (*PS* = 0.43) and lower hydrophobicity (*PS* = 0.38). The positive proteins in the *GPCR_NO* dataset were also slightly more likely to have a longer sequence length than the unlabelled ones (*PS* = 0.59). As GPCRs contain seven transmembrane regions and the positive proteins have a slightly smaller fraction of residues in buried *α*-helices (*PS* = 0.44), the difference in the sequence length likely comes from positive proteins having more extra and intracellular residues. Unlike the amino acids in the transmembrane regions, non-transmembrane residues are likely to be more hydrophilic as they are exposed to the extra and intracellular environments rather than being embedded in a membrane. This likely accounts for the smaller proportion of non-polar and aromatic amino acids in positive proteins and for their lower hydrophobicity. Similarly, the greater proportion of charged and negatively charged amino acids in the positive proteins is likely due to the increased sequence length.

#### Ion Channels

The results from the analysis of the features in the *IonChannel* dataset can be seen in Table 12. The differences between the positive and unlabelled proteins in terms of their amino acid proportions is minimal, with only a small difference in the proportion of non-polar amino acids (*PS* = 0.43). The tendency of the positive proteins to have a slightly smaller proportion of non-polar amino acids can likely be explained by the greater sequence length of the positive proteins (*PS* = 0.61). As the difference in the number of transmembrane helices in positive and unlabelled proteins is minimal (*PS* = 0.48) and the positive proteins have a smaller fraction of residues in buried *α*-helices (*PS* = 0.41), the longer sequence length of the positive proteins can likely be explained by them having more residues in the intra and/or extracellular space. Unlike the amino acids in the transmembrane regions, these residues are likely to be more hydrophilic as they are exposed to the extra and intracellular environments rather than being embedded in a membrane. The positive proteins would therefore have a slightly smaller proportion of non-polar amino acids.

**Table 12:** Results of the feature analysis for the *IonChannel* dataset.

Feature	P-value	PS	Positive Median	Unlabelled Median	Feature	P-value	PS	Positive Median	Unlabelled Median
Alanine	5.21 × 10^−02^	0.44	0.06	0.07	Positively Charged	8.46 × 10^−01^	0.51	0.13	0.13
Arginine	5.91 × 10^−01^	0.52	0.06	0.05	Sequence Length *	4.85 × 10^−04^	0.61	613	509
Asparagine *	9.78 × 10^−04^	0.61	0.04	0.03	PEST Motifs	6.64 × 10^−01^	0.51	0	0
Aspartic Acid	1.57 × 10^−03^	0.60	0.05	0.04	Low Complexity Regions	9.72 × 10^−02^	0.55	3	3
Cysteine	1.36 × 10^−01^	0.45	0.02	0.02	Hydrophobicity	1.90 × 10^−01^	0.46	-0.11	-0.08
Glutamic Acid	2.63 × 10^−01^	0.46	0.06	0.06	Isoelectric Point	8.49 × 10^−01^	0.51	7.38	7.56
Glutamine	1.91 × 10^−03^	0.40	0.03	0.04	Signal Peptide *	1.68 × 10^−10^	0.66	0	0
Glycine	2.50 × 10^−01^	0.46	0.06	0.06	*O*-glycosylation Sites	NA	NA	0	0
Histidine	7.60 × 10^−01^	0.49	0.02	0.02	*N*-glycosylation Sites *	1.02 × 10^−08^	0.68	2	1
Isoleucine	1.54 × 10^−02^	0.58	0.06	0.06	Phosphoserine Sites	2.58 × 10^−03^	0.58	0	0
Leucine *	2.13 × 10^−06^	0.35	0.10	0.11	Phosphothreonine Sites	2.22 × 10^−01^	0.52	0	0
Lysine	2.39 × 10^−01^	0.54	0.05	0.05	Phosphotyrosine Sites	1.59 × 10^−01^	0.53	0	0
Methionine	2.36 × 10^−02^	0.57	0.03	0.02	Total Phosphorylation Sites	9.02 × 10^−03^	0.57	0	0
Phenylalanine	2.79 × 10^−01^	0.46	0.05	0.05	Transmembrane *α*-helices	5.26 × 10^−01^	0.48	4	5
Proline	3.47 × 10^−01^	0.53	0.05	0.05	Exposed *α*-helices	2.85 × 10^−02^	0.43	0.14	0.16
Serine	2.59 × 10^−01^	0.54	0.08	0.07	Buried *α*-helices	3.53 × 10^−03^	0.41	0.27	0.32
Threonine *	2.16 × 10^−04^	0.62	0.05	0.05	*β* Strands	1.26 × 10^−03^	0.60	0.11	0.06
Tryptophan	9.73 × 10^−01^	0.50	0.02	0.02	3’ Untranslated	6.21 × 10^−01^	0.49	0	0
Tyrosine	7.06 × 10^−01^	0.49	0.03	0.03	5’ Untranslated	2.74 × 10^−01^	0.47	0	0
Valine	6.60 × 10^−03^	0.59	0.07	0.06	Nonsynonymous Coding	8.18 × 10^−02^	0.56	4	3
Aliphatic	2.54 × 10^−01^	0.46	0.23	0.23	Synonymous Coding	1.45 × 10^−03^	0.45	0	0
Aromatic	2.67 × 10^−01^	0.46	0.12	0.13	Binary PPIs	2.89 × 10^−01^	0.47	0	0
Charged	8.33 × 10^−01^	0.51	0.23	0.23	Alternative Transcripts	4.44 × 10^−02^	0.56	3	2
Negatively Charged	5.43 × 10^−01^	0.52	0.11	0.10	Paralogues	7.27 × 10^−02^	0.54	0	0
Non-polar	1.59 × 10^−02^	0.42	0.55	0.57	Body Sites Expressed In	4.14 × 10^−01^	0.53	15	15

Shaded features are ones for which the *PS*≥0.5. The amino acid, exposed α-helix, buried α-helix and β strand features are all proportions (e.g. the Alanine feature for a protein is the number of alanine residues in the sequence divided by the sequence length), while all other features are absolute numbers.

Features with significant differences are indicated with an *.

The NAs for the *O*-glycosylation sites are due to no ion channels containing an *O*-glycosylation site.

The increased number of extra and intracellular amino acids could also account for the tendency of the positive proteins to have an increased number of *N*-linked glycosylation (*PS* = 0.68), phosphoserine (*PS* = 0.58) and total phosphorylation sites (*PS* = 0.57). In order to test this, the PS of the three features was tested after accounting for the length of the protein (by dividing the feature value for a protein by the number of residues in its sequence). Following this the positive proteins still had greater values for *N*-linked glycosylation (*PS* = 0.68), phosphoserine (*PS* = 0.56) and total phosphorylation (*PS* = 0.55) sites. However, the effect for the phosphoserine and total phosphorylation sites is now too small to be meaningful, indicating that without the difference in sequence length there would likely be no consequential effect for the phosphoserine or total phosphorylation sites. In contrast to the phosphorylation sites, the PS of the *N*-linked glycosylation sites is the same after controlling for the differences in sequence length, meaning that positive ion channels are likely to have greater *in vivo* half-lives and be more stable. Due to their being more likely to contain a signal peptide, positive ion channels are also more likely to be secreted.

#### Kinases

The results from the analysis of the features in the *Kinase* dataset can be seen in Table 13. Although the results indicate that there are significant differences between the positive and unlabelled proteins, the substantial differences in their compositions, specifically the much larger proportion of tyrosine kinases in the positive proteins (Table 14), are potentially influencing the results. The significant differences seen for the *Kinase* dataset could then simply be a reflection of the differences between serine/threonine and tyrosine kinases. Although the presence of kinases of an unknown type complicates this generalisation somewhat, in general their feature values closely follow those of the serine/threonine kinases, not the tyrosine ones. They are therefore likely to be at best neutral with regards to the differences between the serine/threonine and tyrosine kinases.

**Table 13:** Results of the feature analysis for the *Kinase* dataset.

Feature	P-value	PS	Positive Median	Unlabelled Median	Feature	P-value	PS	Positive Median	Unlabelled Median
Alanine	1.19 × 10^−02^	0.42	0.06	0.07	Positively Charged	3.66 × 10^−03^	0.41	0.14	0.15
Arginine	6.76 × 10^−02^	0.44	0.06	0.06	Sequence Length	1.21 × 10^−01^	0.55	682	587
Asparagine	3.38 × 10^−03^	0.59	0.04	0.03	PEST Motifs	4.16 × 10^−02^	0.44	0	0
Aspartic Acid	2.39 × 10^−02^	0.57	0.05	0.05	Low Complexity Regions	3.05 × 10^−01^	0.47	2	2
Cysteine	3.63 × 10^−03^	0.59	0.02	0.02	Hydrophobicity	4.61 × 10^−02^	0.56	-0.35	-0.38
Glutamic Acid	4.14 × 10^−01^	0.47	0.07	0.07	Isoelectric Point	4.59 × 10^−03^	0.41	6.87	7.12
Glutamine	1.32 × 10^−02^	0.42	0.04	0.04	Signal Peptide *	2.74 × 10^−10^	0.63	0	0
Glycine	1.67 × 10^−01^	0.54	0.07	0.06	*O*-glycosylation Sites	2.64 × 10^−01^	0.50	0	0
Histidine	4.38 × 10^−01^	0.48	0.03	0.03	*N*-glycosylation Sites *	3.84 × 10^−12^	0.64	0	0
Isoleucine	8.21 × 10^−02^	0.56	0.05	0.05	Phosphoserine Sites	1.78 × 10^−01^	0.54	2	1
Leucine	8.32 × 10^−01^	0.49	0.10	0.10	Phosphothreonine Sites	2.94 × 10^−02^	0.56	1	0
Lysine	2.25 × 10^−01^	0.46	0.06	0.06	Phosphotyrosine Sites *	3.49 × 10^−18^	0.74	2	0
Methionine	1.25 × 10^−01^	0.55	0.02	0.02	Total Phosphorylation Sites *	4.05 × 10^−08^	0.67	8	3
Phenylalanine	2.42 × 10^−01^	0.54	0.04	0.04	Transmembrane *α*-helices *	4.34 × 10^−08^	0.62	0	0
Proline	2.82 × 10^−01^	0.47	0.06	0.06	Exposed *α*-helices *	9.94 × 10^−06^	0.36	0.10	0.13
Serine	3.86 × 10^−02^	0.43	0.07	0.07	Buried *α*-helices	9.53 × 10^−02^	0.45	0.13	0.15
Threonine	6.87 × 10^−02^	0.56	0.05	0.05	*β* Strands *	5.12 × 10^−10^	0.70	0.17	0.13
Tryptophan *	7.12 × 10^−05^	0.63	0.01	0.01	3’ Untranslated	2.16 × 10^−01^	0.54	2	1
Tyrosine *	3.69 × 10^−04^	0.61	0.03	0.03	5’ Untranslated	2.41 × 10^−01^	0.54	2	1
Valine	8.16 × 10^−02^	0.56	0.06	0.06	Nonsynonymous Coding	5.29 × 10^−03^	0.59	24	17
Aliphatic	1.44 × 10^−01^	0.55	0.21	0.21	Synonymous Coding	4.58 × 10^−01^	0.52	0	0
Aromatic	1.83 × 10^−03^	0.60	0.11	0.10	Binary PPIs	1.20 × 10^−02^	0.58	2	1
Charged	6.80 × 10^−02^	0.44	0.26	0.27	Alternative Transcripts	2.12 × 10^−01^	0.54	4	3
Negatively Charged	6.31 × 10^−01^	0.52	0.12	0.12	Paralogues	9.48 × 10^−01^	0.50	0	0
Non-polar	7.77 × 10^−02^	0.56	0.53	0.53	Body Sites Expressed In	8.89 × 10^−03^	0.58	33	31

Shaded features are ones for which the *PS*≥0.5. The amino acid, exposed α-helix, buried α-helix and β strand features are all proportions (e.g. the Alanine feature for a protein is the number of alanine residues in the sequence divided by the sequence length), while all other features are absolute numbers.

Features with significant differences are indicated with an *.

**Table 14:** Division of positive and unlabelled kinases by type.

	Serine/Threonine	Tyrosine	Atypical	Unknown
Entire Dataset	390 (59%)	90 (14%)	27 (4%)	154 (23%)
Unlabelled Proteins	355 (63%)	50 (9%)	26 (5%)	136 (24%)
Unlabelled Proteins With Positive Similarity >0.5	56 (52%)	33 (31%)	1 (1%)	17 (16%)
Unlabelled Proteins With Positive Similarity ≥0.75	16 (33%)	26 (53%)	0 (0%)	7 (14%)
Positive Proteins	35 (37%)	40 (43%)	1 (1%)	18 (19%)

The distribution of proteins in the entire *Kinase* dataset, all unlabelled proteins, misclassified unlabelled proteins and all positive proteins by kinase type. Unknown kinases are ones where it is not known whether they are Serine/Threonine, Tyrosine or atypical kinases.

The influence of the differences between kinase types was evaluated by creating two new datasets. The *Kinase_TK* dataset was constructed from the *Kinase* dataset by removing all positive proteins that were not tyrosine kinases, while the *Kinase_NTK* dataset was constructed from the *Kinase* dataset by removing all positive proteins that were tyrosine kinases. Both of these datasets had their features analysed in terms of significance and effect size using. A comparison between the features that are significant in each of the three datasets can be seen in Table 15. For each feature, the deviation of the effect size from 0.5 in the *Kinase* dataset can be seen to be between the deviations for the *Kinase_TK* and *Kinase_NTK* datasets. However, if the positive serine/threonine and tyrosine kinases shared similar properties, then the features with the greatest deviations in effect size in the *Kinase* dataset would be expected to have the greatest deviations in the *Kinase_TK* and *Kinase_NTK* datasets. The pattern of deviations therefore indicates that there are distinct differences between the positive serine/threonine kinases and the positive tyrosine ones. Additionally, the positive tyrosine kinases can be seen to be dominating the effects seen in the *Kinase* dataset, as the *Kinase_TK* dataset shows very large deviations for those features that are significant in the *Kinase* dataset while the *Kinase_NTK* dataset has very small deviations for them. Additionally, of the nine significant features in the *Kinase* dataset, all nine were found to be significant in the *Kinase_TK* dataset, while none were significant in the *Kinase_NTK* dataset. These results indicate that the differences between the positive and unlabelled proteins in the *Kinase* dataset are highly likely to be a consequence of the makeup of the dataset, rather than a true reflection of the properties that make a kinase a suitable drug target.

**Table 15:** Comparison of the feature effect sizes across the three datasets of kinases.

Feature	Kinase	Kinase_NTK	Kinase_TK
Phosphotyrosine Sites	* 0.24	0.07	* 0.48
β Strands	* 0.20	0.08	* 0.35
Total Phosphorylation Sites	* 0.17	0.08	* 0.30
Exposed *α*-helices	*-0.14	0.03	* -0.37
*N*-Glycosylation Sites	* 0.14	0.03	* 0.29
Signal Peptide	* 0.13	0.02	* 0.27
Tryptophan	* 0.13	0.03	* 0.25
Transmembrane *α*-helices	* 0.12	0.01	* 0.26
Tyrosine	* 0.11	0.03	* 0.22
Aromatic	0.10	0.08	0.12
Arginine	0.09	0.04	* 0.16
Cysteine	0.09	0.05	0.15
Positively Charged	-0.09	0.00	* -0.22
Isoelectric Point	-0.09	-0.04	* -0.16
Nonsynonymous Coding	0.09	0.11	0.06
Body Sites Expressed In	0.08	0.11	0.05
Alanine	-0.08	-0.09	-0.07
Glutamine	-0.08	-0.03	-0.15
Binary PPIs	0.08	0.12	0.02
Aspartic Acid	0.07	* 0.14	-0.01

Effect size deviation from ***PS* = 0.5** (no effect) for the twenty features with the largest effect size in the ***Kinase*** dataset. A negative value indicates that the positive proteins have smaller values than the unlabelled ones, while a positive value indicates that they have greater ones.

Features with significant differences in a dataset are indicated with an *.

Despite the entire set of kinases being unsuitable for analysis, it is possible that informative differences can be found by comparing positive tyrosine kinases with unlabelled ones. However, in addition to the differences between the serine/threonine and tyrosine kinases, the *Kinase* dataset also has a biased set of positive tyrosine kinases. Although it may be hypothesised that this bias would be due to a preference for receptor tyrosine kinases, due to drug targets being predominantly membrane bound, the fraction of receptor tyrosine kinases in the positive proteins and in the set of all tyrosine kinases is very similar. Rather, the source of the bias comes from the specific disease that the drugs targeting the tyrosine kinases are intended to treat: cancer. Of the forty positive tyrosine kinases, thirty-four are the target of an antineoplastic drug, while a further three are causally implicated in cancer (and therefore in the *Cancer* dataset). However, only four of the fifty unlabelled tyrosine kinases are causally implicated in cancer. Any comparison of positive and unlabelled tyrosine kinases is therefore more of a comparison between those tyrosine kinases that have been implicated in cancer and those that have not.

While the positive serine/threonine kinases have no biases as evident as those of the tyrosine kinases, there are very few of them. Additionally, these kinases may be unrepresentative in that they may have been selected as early targets for specific reasons, e.g. properties that they possess, that will not extrapolate to future targets. Therefore, until the set of positive kinases is more representative, or the set of positive serine/threonine kinases increases in size, it will be difficult to get an accurate picture of the properties that make a general kinase a suitable drug target, other than the inhibition of phosphoryl group transfer from nucleotides.

#### Proteases

The results from the analysis of the features in the *Protease* dataset can be seen in Table 16. Although these results indicate that significant differences between the positive and unlabelled proteins can be found, care must be taken due to the composition of the set of positive proteins. As 61% of positive proteins are metallo proteases it is possible that differences in the datasets are reflecting differences between a specific subset of the metallo proteases (the positive proteins) and proteases in general (the unlabelled ones). This is of particular concern due to the high level of similarity within the positive metallo proteases and low level of similarity between the positive metallo and non-metallo proteases. In order to evaluate the effect of this subpopulation of positive metallo proteases, two further datasets were constructed. The *Protease_MP* dataset was constructed from the *Protease* dataset by removing all positive proteins that were not metallo proteases, while the *Protease_NMP* dataset was constructed from the *Protease* dataset by removing all positive proteins that were metallo proteases. Both of these datasets had their features analysed in terms of significance and effect size. A comparison between the features that are significant in each of the three datasets can be seen in Table 17. For each feature, the deviation of the effect size from 0.5 in the *Protease* dataset can also be seen to be between the deviations for the *Protease_MP* and *Protease_NMP* datasets, except for the glutamine proportion and synonymous coding variants. However, if the positive metallo proteases and non-metallo proteases shared similar properties, then the features with the greatest effect size deviations in the *Protease* dataset would be expected to have the greatest deviations in the *Protease_MP* and *Protease_NMP* datasets. The differing pattern of deviations therefore indicates that there are distinct differences between the positive metallo proteases and the positive non-metallo ones. This can be seen most clearly in the proportion of cysteine in the proteins, with both the *Protease_MP* and *Protease_NMP* datasets having sizable effects for it, but with the positive proteins having a greater proportion of cysteine in the *Protease_NMP* dataset and a smaller proportion in the *Protease_MP* one. Additionally, of the ten significant features in the *Protease* dataset, eight were found to be significant in the *Protease_MP* dataset, while only one was significant in the *Protease_NMP* dataset. These results indicate that the differences in the *Protease* dataset are likely reflecting the differences between the positive metallo proteases and the unlabelled proteases, rather than capturing properties of protease drug targets in general.

**Table 16:** Results of the feature analysis for the *Protease* dataset.

Feature	P-value	PS	Positive Median	Unlabelled Median	Feature	P-value	PS	Positive Median	Unlabelled Median
Alanine	8.99 × 10^−02^	0.57	0.07	0.06	Positively Charged	6.29 × 10^−01^	0.52	0.14	0.13
Arginine	2.25 × 10^−02^	0.59	0.06	0.05	Sequence Length	6.61 × 10^−01^	0.48	478	497
Asparagine	7.89 × 10^−01^	0.49	0.04	0.04	PEST Motifs	8.80 × 10^−02^	0.44	0	0
Aspartic Acid *	5.34 × 10^−05^	0.66	0.06	0.05	Low Complexity Regions	4.34 × 10^−01^	0.47	1	2
Cysteine *	3.71 × 10^−05^	0.34	0.01	0.03	Hydrophobicity	2.23 × 10^−02^	0.41	-0.39	-0.30
Glutamic Acid	1.70 × 10^−01^	0.45	0.05	0.06	Isoelectric Point	6.88 × 10^−01^	0.48	6.97	7.06
Glutamine *	3.56 × 10^−04^	0.36	0.04	0.04	Signal Peptide *	8.10 × 10^−04^	0.62	1	0
Glycine	1.23 × 10^−01^	0.56	0.08	0.08	*O*-glycosylation Sites *	6.08 × 10^−08^	0.57	0	0
Histidine	3.58 × 10^−01^	0.46	0.03	0.03	*N*-glycosylation Sites	1.81 × 10^−02^	0.59	1	0
Isoleucine	2.03 × 10^−01^	0.45	0.04	0.05	Phosphoserine Sites	2.21 × 10^−01^	0.47	0	0
Leucine	8.30 × 10^−03^	0.40	0.09	0.09	Phosphothreonine Sites	3.58 × 10^−01^	0.48	0	0
Lysine	2.98 × 10^−01^	0.46	0.05	0.05	Phosphotyrosine Sites	7.18 × 10^−01^	0.49	0	0
Methionine	9.92 × 10^−01^	0.50	0.02	0.02	Total Phosphorylation Sites	5.74 × 10^−01^	0.48	0	0
Phenylalanine *	1.47 × 10^−04^	0.65	0.04	0.04	Transmembrane *α*-helices	7.23 × 10^−01^	0.51	0	0
Proline	3.20 × 10^−01^	0.54	0.06	0.06	Exposed *α*-helices	4.04 × 10^−01^	0.47	0.07	0.09
Serine *	1.43 × 10^−05^	0.33	0.06	0.07	Buried *α*-helices	9.19 × 10^−01^	0.50	0.11	0.11
Threonine	1.17 × 10^−01^	0.56	0.05	0.05	*β* Strands	9.23 × 10^−02^	0.57	0.21	0.17
Tryptophan	1.32 × 10^−01^	0.56	0.02	0.02	3’ Untranslated	5.15 × 10^−01^	0.48	0	0
Tyrosine *	2.83 × 10^−06^	0.68	0.04	0.03	5’ Untranslated	1.78 × 10^−01^	0.45	0	0
Valine	2.80 × 10^−03^	0.38	0.06	0.06	Nonsynonymous Coding	3.23 × 10^−01^	0.54	13	12
Aliphatic *	2.41 × 10^−05^	0.33	0.19	0.21	Synonymous Coding	2.53 × 10^−02^	0.57	0	0
Aromatic *	3.36 × 10^−06^	0.68	0.13	0.12	Binary PPIs	8.77 × 10^−01^	0.51	0	0
Charged	5.48 × 10^−01^	0.52	0.25	0.25	Alternative Transcripts	3.39 × 10^−01^	0.46	2	2
Negatively Charged	1.69 × 10^−01^	0.55	0.11	0.11	Paralogues	1.22 × 10^−01^	0.46	0	0
Non-polar	1.33 × 10^−01^	0.56	0.56	0.55	Body Sites Expressed In	5.70 × 10^−01^	0.52	25	25

Shaded features are ones for which the ***PS*≥0.5**. The amino acid, exposed α-helix, buried α-helix and β strand features are all proportions (e.g. the Alanine feature for a protein is the number of alanine residues in the sequence divided by the sequence length), while all other features are absolute numbers.

Features with significant differences are indicated with an *.

**Table 17:** Comparison of the feature effect sizes across the three datasets of proteases.

Feature	Protease	Protease_NMP	Protease_MP
Tyrosine	* 0.18	0.12	* 0.22
Aromatic	* 0.18	-0.02	* 0.31
Serine	* -0.17	-0.02	* -0.27
Aliphatic	* -0.17	-0.10	* -0.21
Cysteine	* -0.16	0.11	* -0.34
Aspartic Acid	* 0.16	0.04	* 0.23
Phenylalanine	* 0.15	-0.06	* 0.29
Glutamine	* -0.14	-0.14	-0.14
Valine	-0.12	-0.04	* -0.17
Signal Peptide	* 0.12	0.07	* 0.15
Leucine	-0.10	-0.13	-0.09
Hydrophobicity	-0.09	0.00	-0.15
Arginine	0.09	0.14	0.06
*N*-Glycosylation Sites	0.09	0.06	0.10
*O*-Glycosylation Sites	* 0.07	* 0.17	0.01
Synonymous Coding	0.07	0.09	0.07
Alanine	0.07	0.02	0.10
*β* Strands	0.07	0.16	0.01
Threonine	0.06	0.14	0.01
Glycine	0.06	0.14	0.01

Effect size deviation from ***PS* = 0.5** (no effect) for the twenty features with the largest effect size in the ***Protease*** dataset. A negative value indicates that the positive proteins have smaller values than the unlabelled ones, while a positive value indicates that they have greater ones.

Features with significant differences in a dataset are indicated with an *.

As the metallo proteases (both positive and unlabelled) appear to cluster together, one method for overcoming the problems with the *Protease* dataset would be to compare positive metallo proteases to unlabelled ones. The same could then be done for non-metallo proteases, or alternatively for further subsets of the *Protease* dataset (e.g. serine proteases). However, this approach would be problematic due to the small size of the set of positive proteins, as there are only thirty-six positive metallo proteases, and the possible bias towards certain metallo proteases having been selected as early targets due to their specific properties or the simplicity of targeting them. Therefore, more positive proteases are needed in order to accurately determine the properties of protease drug targets.

#### Target Predictions

After optimising the parameters for a dataset, a RF was trained on the dataset and used to classify the proteins in it. The classification for an individual protein consisted of two parts: the RF’s weighted vote for the unlabelled class and its weighted vote for the positive class. From these two values the positive similarity of a protein can be calculated as the fraction of the RF’s total vote for the positive class. This similarity can be thought of as the confidence of the RF in its prediction, and can therefore be used as a measure of a protein’s drug target likeness. The final classification of a protein can then be determined from its similarity by defining a cutoff, such that proteins are classified as positive only if they have a positive similarity above the cutoff. A cutoff of 0.5 was used here, as a similarity greater than this indicates that the majority of the RF’s vote was for the positive class.

#### All Proteins

**Fig 1:**
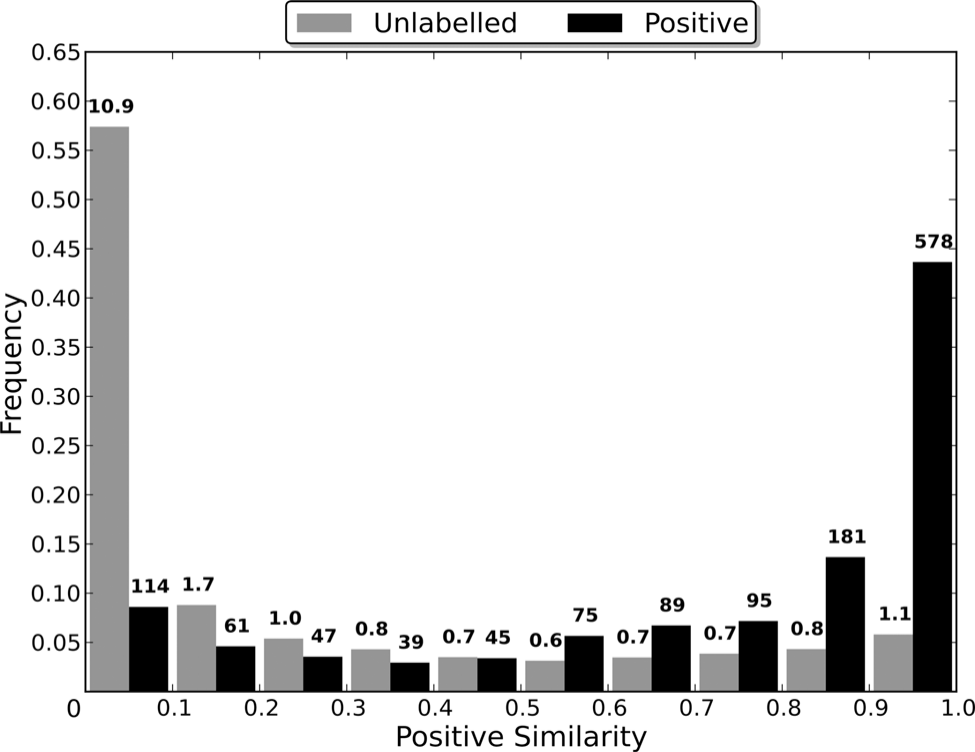
Weighted predictions of the proteins in the *AllTargets* dataset. The positive similarity of a given protein is equal to the fraction of the forest’s votes that are for the positive class. The values over the bars indicate the number of proteins in the bin (in raw numbers for the positive (black) bars and in thousands for the unlabelled (grey) bars). The ***AllTargets*** dataset contained 18919 unlabelled proteins and 1324 positive ones.

The best combination of parameters and feature set for classifying the proteins in the *AllTargets* dataset was *numberTrees* = 1000, *mtry* = 5, a weight of 110 given to each observation in the in positive class, a random seed of 3079726279227244970 and forty features out of the original 105 (S3 Supplementary Information). The positive similarity of the proteins in the *AllTargets* dataset can be seen in Fig. 1. Using a cutoff of 0.5, the RF’s predicted classifications are shown in Table 18.

**Table 18:** Random Forest predicted classifications for all proteins.

Positive Observations				Unlabelled Observations				G Mean
Total	TPs	FNs	Sensitivity	Total	TNs	FPs	Specificity	
1324	1018	306	0.77	18919	15021	3898	0.79	0.78

A G mean of 0.78 indicates that the RF had some difficulty classifying proteins in the *AllTargets* dataset. While this may be due to the inadequacy of RFs for the task, the performance of the RFs on other datasets indicates that it is more likely due to the *AllTargets* dataset itself. As the *AllTargets* dataset contains a heterogeneous set of proteins, due to there being no additional membership criteria, the distinction between positive and unlabelled proteins may be more difficult to make, as unlabelled proteins in one family may overlap with positive proteins in others. Additionally, proteins from smaller families will likely form poorly defined clusters. These proteins will therefore be more difficult to classify correctly, and will also increase the difficulty involved in the classification of the proteins in the larger families. In order to test this theory, two new datasets were created from the *AllTargets* dataset. The first dataset, *LargeFamilies*, consisted of all proteins in the *GPCR, IonChannel, Kinase* and *Protease* datasets, and the second dataset, *SmallFamilies*, consisted of all proteins in *AllTargets-LargeFamilies*. RFs were optimised for these two datasets and used to classify the proteins in the dataset that they were trained on. The G mean of the RF optimised for the *LargeFamilies* dataset was 0.81, and the G mean of the RF optimised for the *SmallFamilies* dataset was 0.76. As expected, the proteins in the smaller families were more difficult to classify, and the proteins in the larger families were classified with a G mean greater than that of the *AllTargets* dataset. These results indicate that it is likely to be the combination of the protein families that makes accurate classifications more difficult, and that including smaller families is detrimental to the classification of proteins in general.

#### Cancer Proteins

**Fig 2:**
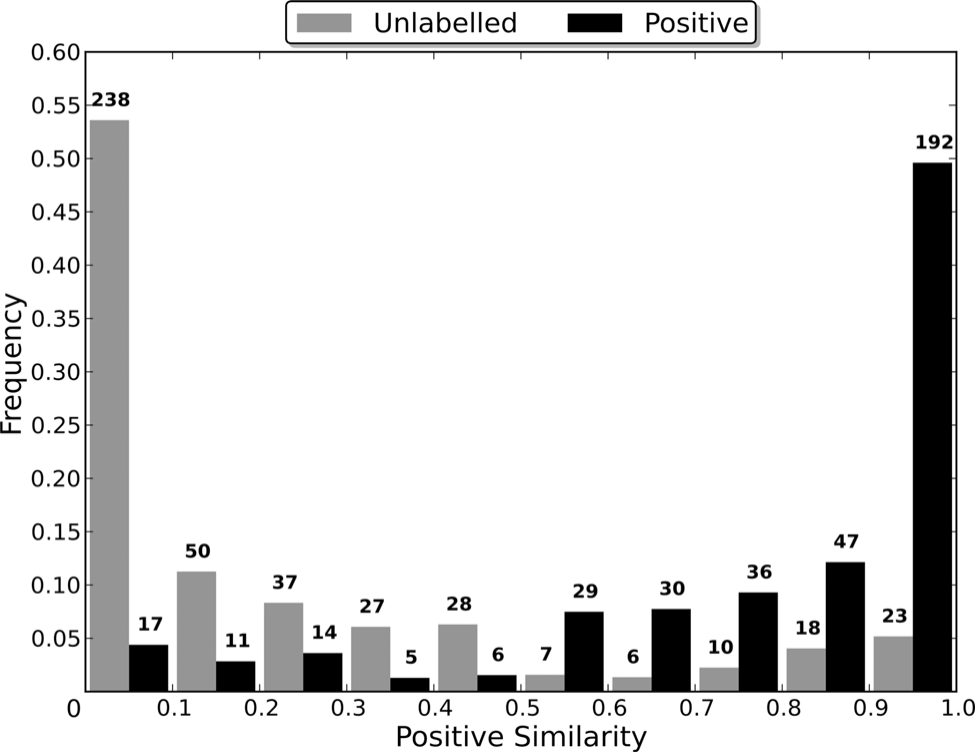
Weighted predictions of the proteins in the *Cancer* dataset.

The best combination of parameters and feature set for classifying the proteins in the *Cancer* dataset was *numberTrees* = 1000, *mtry* = 5, a weight of 1.3 given to each observation in the in positive class, a random seed of—4923865346116695007 and thirty-six features out of the original 105 (S3 Supplementary Information). The positive similarity of the proteins in the *Cancer* dataset can be seen in Fig. 2. Using a cutoff of 0.5, the RF’s predicted classifications are shown in Table 19.

**Table 19:** Random Forest predicted classifications for Cancer proteins.

Positive Observations				Unlabelled Observations				G Mean
Total	TPs	FNs	Sensitivity	Total	TNs	FPs	Specificity	
387	334	53	0.86	444	380	64	0.86	0.86

As 55% of positive proteins are membrane bound, compared to 11% of unlabelled ones, it would be expected that a substantial fraction of the misclassified unlabelled proteins are also membrane bound. This was found to be the case, with 55% of unlabelled proteins with a positive similarity >0.5 being membrane bound, and 65% of the unlabelled proteins most likely to be suitable targets, those with positive similarity ≥0.75, being membrane bound. This tendency to be membrane bound is also reflected in the function of the forty-six unlabelled proteins with positive similarity ≥0.75, as a large fraction of them are membrane bound receptors involved in signal transduction. Many of the proteins are also putative proto-oncogenes or tumour suppressors, and are often involved in a process or processes that can contribute to the distinguishing characteristics of cancer. For example, the unlabelled protein with the greatest positive similarity was protein patched homolog 1 (PTCH1) (UniProt accession Q13635). In addition to being a known tumour suppressor, PTCH1 is a receptor for hedgehog ligands, which are involved in proliferation and differentiation during embryogenesis [64]. Even when a direct connection between the protein and cancer is speculative or unknown, a connection between them can often be hypothesised. For example, although sodium-dependent phosphate transport protein 2B (UniProt accession O95436) has no clear oncogenic or tumour suppression function, it is regulated by epidermal growth factor [65], the expression of which is often altered in cancerous cells as part of their achieving unregulated growth. In addition to the proteins that can be causatively linked to cancer, there are others, such as solute carrier family 45 member 3 (UniProt accession Q96JT2) which are differentially expressed in cancer but not presently believed to be drivers of cancer [66,67]. While connections between the proteins and cancer provide some validation for the usefulness of the misclassified unlabelled proteins as antineoplastic targets, at least one, programmed cell death 1 ligand 1 (UniProt accession Q9NZQ7), is known to be the target of a compound currently undergoing phase II clinical trials as an antineoplastic drug (MPDL3280A). All unlabelled proteins in the *Cancer* dataset predicted to be positive can be found in S4 Supplementary Information. These proteins are those that have been causatively linked to cancer, without currently (2014) being used as an antineoplastic target, and appear most suitable for consideration as future antineoplastic drug targets.

#### GPCRs

**Fig 3:**
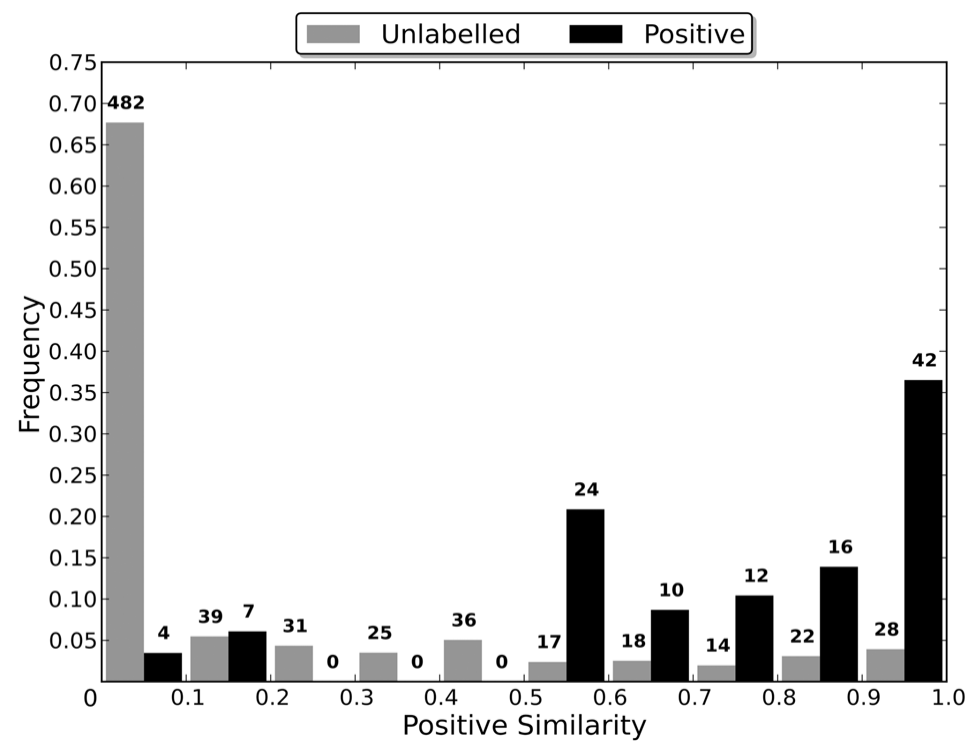
Weighted predictions of the proteins in the *GPCR* dataset.

The best combination of parameters and feature set for classifying the proteins in the *GPCR* dataset was *numberTrees* = 4000, *mtry* = 5, a weight of 12 given to each observation in the in positive class, a random seed of -4568194888819162440 and forty-two features out of the original 105 (S3 Supplementary Information). The positive similarity of the proteins in the *GPCR* dataset can be seen in Fig. 3. Using a cutoff of 0.5, the RF’s predicted classifications are shown in Table 20.

**Table 20:** Random Forest predicted classifications for GPCR proteins.

Positive Observations				Unlabelled Observations				G Mean
Total	TPs	FNs	Sensitivity	Total	TNs	FPs	Specificity	
115	104	11	0.90	712	613	99	0.86	0.88

As with the results of the analysis of the features in the *GPCR* dataset, the distribution of the positive similarities of the proteins in the dataset is likely to be heavily skewed by the presence of the odorant/olfactory GPCRs. Of the 421 odorant/olfactory GPCRs in the dataset, 419 were given a positive similarity below 0.1, with all 421 having a positive similarity below 0.5. In order to assess the impact on the classifications of including the odorant/olfactory GPCRs in the dataset, a new dataset, *GPCR_NO*, was constructed from all proteins in the *GPCR* dataset that are not odorant/olfactory receptors. The best combination of parameters and feature set for classifying the proteins in the *GPCR_NO* dataset was *numberTrees* = 4000, *mtry* = 5, a weight of 3.6 given to each observation in the in positive class, a random seed of -251746180866936552 and forty-seven features out of the original 105 (S3 Supplementary Information).

**Fig 4:**
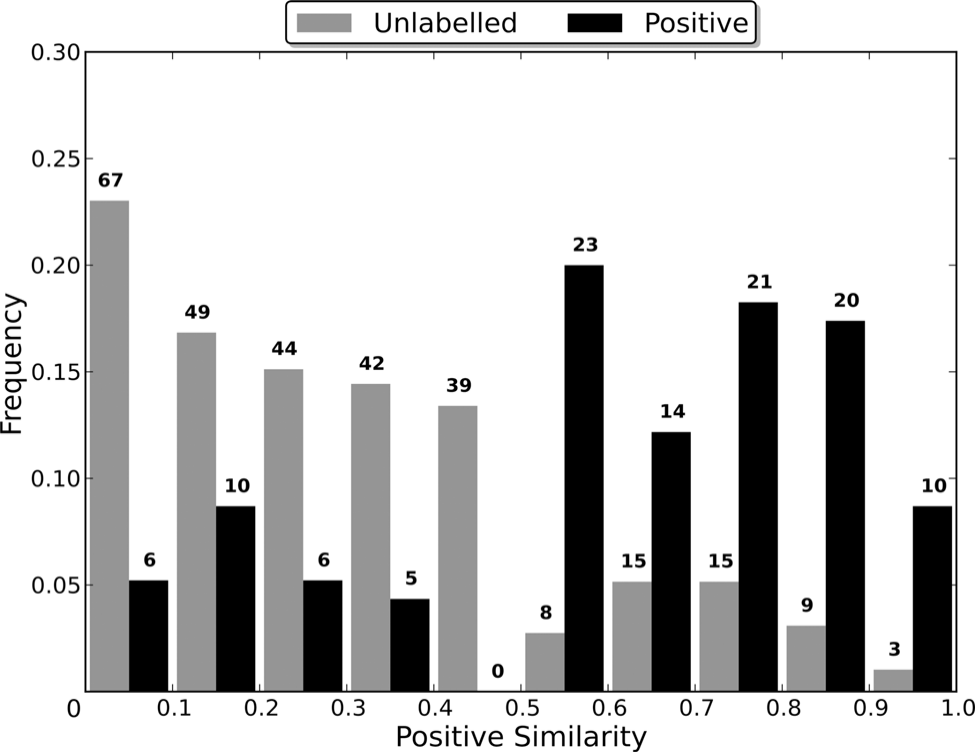
Weighted predictions of the proteins in the *GPCR_NO* dataset.

The lower G mean of the RF trained on the *GPCR_NO* dataset indicates that the dissimilarities between the positive and unlabelled proteins are not as great as the results generated using the *GPCR* dataset would purport to show (Table 21). The positive and unlabelled GPCRs are in fact quite similar, once the odorants are removed, as can be seen from the large overlap and relatively low frequencies in their positive similarities (Fig. 4) and the small effect size of their differences (Table 11).

**Table 21:** A comparison of the predictions of the non-odorant GPCRs.

Dataset Trained On	Positive Observations				Unlabelled Observations				G Mean
	Total	TPs	FNs	Sensitivity	Total	TNs	FPs	Specificity	
*GPCR*	115	104	11	0.90	291	241	99	0.71	0.74
*GPCR_NO*	115	88	27	0.77	291	241	50	0.83	0.80

Predictions were made by the optimised RF trained on the *GPCR* dataset and the one trained on the *GPCR_NO* dataset.

Using a cutoff of 0.5, substantial differences can be seen in the classifications of the non-odorant GPCRs by the RFs trained on the *GPCR* and *GPCR_NO* datasets (Tables 10 and 11). Although no odorant receptors were misclassified by the RF trained on the *GPCR* dataset, removing the odorants from the dataset led to 49 fewer unlabelled proteins being misclassified as positive. Although this may appear counterintuitive, it is in fact unsurprising. This is because the presence of the large subpopulation of unlabelled odorants allows the weight given to the positive observations to be increased, thereby improving the sensitivity of the RF at the cost of increasing the number of misclassified unlabelled proteins. As only non-odorant unlabelled proteins will be misclassified, due to the dissimilarity between the odorants and positive proteins, the resultant decrease in specificity will be small and can be more than compensated for by the increase in sensitivity. Therefore, removing the odorants will not only negate the artificial boost to the specificity that they provide, but also necessitate a decrease in the weight given to the positive proteins. The need for this decrease can be seen in the fact that using a weight of 12, as was done for the *GPCR* dataset, causes a large number of unlabelled non-odorants to be misclassified (Table 10). As the G mean of the RF trained on the *GPCR_NO* dataset is substantially more sensitive to misclassified unlabelled proteins, due to the smaller number of unlabelled proteins in the dataset, the number of misclassified unlabelled proteins must be brought down in order to achieve a respectable G mean. However, the increase in specificity that this provides will be accompanied by a sizeable decrease in sensitivity, and therefore a lower G mean.

Of the twenty-three unlabelled proteins with the greatest likelihood of being suitable drug targets, those with positive similarity ≥0.75, 15 are class A GPCRs, 7 are class B and 1 is class C. Irrespective of class, the GPCRs are predominantly expressed in the brain and the central nervous system. In terms of the ligands of the misclassified unlabelled proteins, seven of the twenty-three are orphan receptors with no known ligand, while the remainder are predominantly receptors for neurotransmitters and neuropeptides (in line with their tendency to be expressed in the brain). All unlabelled proteins in the *GPCR_NO* dataset predicted to be positive can be found in SI3.

#### Ion Channels

**Fig 5:**
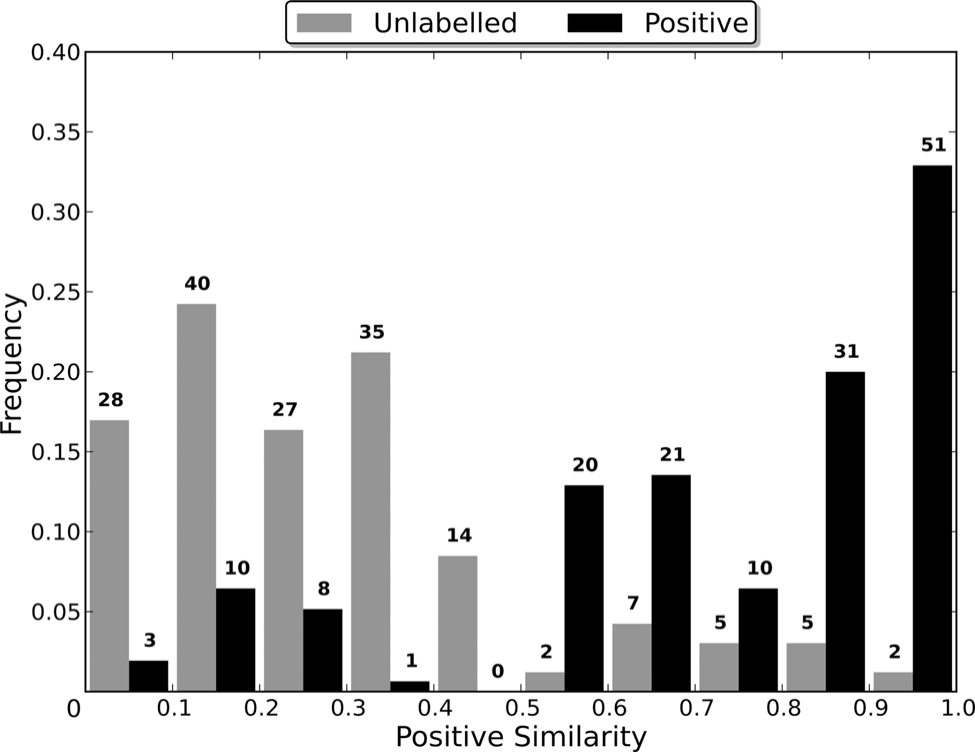
Weighted predictions of the proteins in the *IonChannel* dataset.

The best combination of parameters and feature set for classifying the proteins in the *IonChannel* dataset was *numberTrees* = 1000, *mtry* = 10, a weight of 1.2 given to each observation in the in positive class, a random seed of 2641231349290994133 and forty features out of the original 105 (S3 Supplementary Information). The positive similarity of the proteins in the *IonChannel* dataset can be seen in Fig. 5. The distribution of the proteins in the *IonChannel* dataset likely indicates that there is a strong similarity between the positive and unlabelled proteins, as there are no particularly large peaks in any of the bins more extreme bins (0.0–0.1 and 0.9–1.0). Using a cutoff of 0.5, the RF’s predicted classifications are shown in Table 22.

**Table 22:** Random Forest predicted classifications for Ion Channel proteins.

Positive Observations				Unlabelled Observations				G Mean
Total	TPs	FNs	Sensitivity	Total	TNs	FPs	Specificity	
155	133	22	0.86	165	144	21	0.87	0.87

Of the ten unlabelled proteins with the greatest likelihood of being suitable drug targets, those with positive similarity ≥0.75, six are known to be voltage-gated, three ligand-gated and one of unknown gating. Of the voltage-gated channels, two are selective for calcium, three for potassium and one for sodium, with the potassium channels both being inward rectifying ones. All three ligand gated channels were selective for cations, with the ligands being zinc for one channel and serotonin for the other two. All unlabelled proteins in the *IonChannel* dataset predicted to be positive can be found in SI3.

#### Kinases

**Fig 6:**
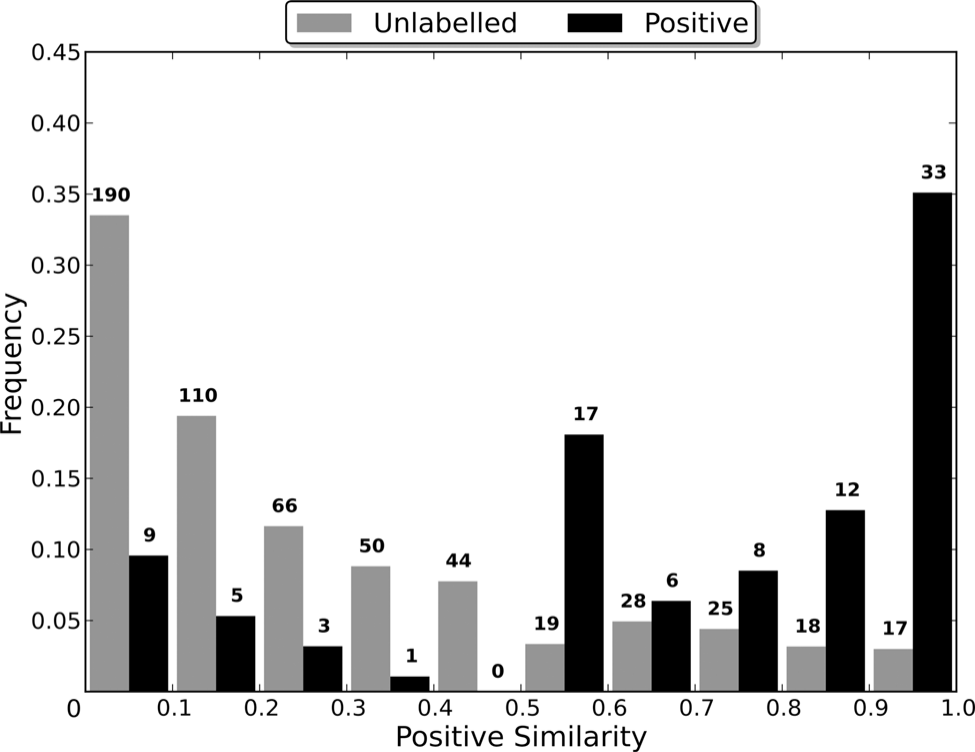
Weighted predictions of the proteins in the *Kinase* dataset.

The best combination of parameters and feature set for classifying the proteins in the *Kinase* dataset was *numberTrees* = 1000, *mtry* = 5, a weight of 23 given to each observation in the in positive class, a random seed of -6712145332927501964 and thirty-two features out of the original 105 (S3 Supplementary Information). The positive similarity of the proteins in the *Kinase* dataset can be seen in Fig. 6. The distribution of the proteins in the *Kinase* dataset likely indicates that there is a strong similarity between the positive and unlabelled proteins, as there are no particularly large peaks in any of the bins more extreme bins (0.0–0.1 and 0.9–1.0). Using a cutoff of 0.5, the RF’s predicted classifications are shown in Table 23.

**Table 23:** Random Forest predicted classifications for Kinases.

Positive Observations				Unlabelled Observations				G Mean
Total	TPs	FNs	Sensitivity	Total	TNs	FPs	Specificity	
94	76	18	0.81	567	460	107	0.81	0.81

Two clear trends can be discerned by looking at the types of the kinases in the *Kinase* dataset (Table 14). Firstly, atypical (i.e. not Ser, Thr or Tyr) kinases make poor targets. Of the twenty-seven kinases known to be atypical, only one is the target of an approved drug. Similarly, only one unlabelled atypical kinase is misclassified as positive, although with a positive similarity <0.75. There are therefore no atypical kinases amongst the forty-nine unlabelled kinases that are most likely to be suitable targets, those with positive similarity ≥0.75. The second clear trend is the preferential targeting of tyrosine kinases. Despite only 14% of all kinases of known type being tyrosine kinases, they comprise 43% of the positive kinases of known type. Additionally, if the misclassified unlabelled proteins are included, then 73 of the 90 kinases that are known to be tyrosine kinases are targets or are believed to be suitable future targets. Further evidence of the disproportionate importance of tyrosine kinases as drug targets can be seen in the fact that 31% of the misclassified unlabelled proteins, and 53% of the unlabelled proteins with positive similarity ≥0.75, are tyrosine kinases.

In addition to the type of the kinase, the misclassified unlabelled proteins share with the positive proteins a tendency to be membrane bound. Although 17% of unlabelled and 34% of positive proteins are membrane proteins, 24% of the unlabelled proteins with positive similarity >0.5 are. However, of the unlabelled proteins with positive similarity ≥0.75, twenty (41%) are membrane bound. This further highlights the influence of tyrosine kinases on the prediction of kinase drug targets, as nineteen of the twenty misclassified membrane bound unlabelled proteins with positive similarity ≥0.75 are receptor tyrosine kinases. When considering receptor and non-receptor tyrosine kinases separately, the receptor tyrosine kinases make up the largest fraction of the misclassified unlabelled proteins with positive similarity ≥0.75. The importance of being membrane bound to the likelihood of a kinase being a suitable drug target is therefore likely to be more of a reflection of the importance of being a receptor tyrosine kinase. All unlabelled proteins in the *Kinase* dataset predicted to be positive can be found in S3 Supplementary Information.

#### Proteases

**Fig 7:**
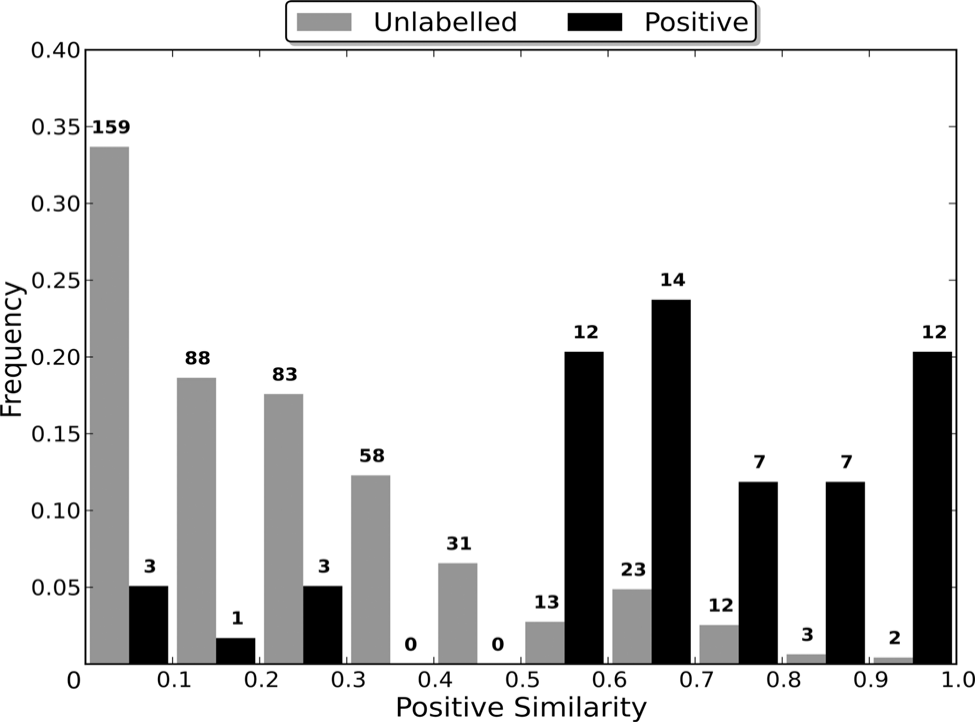
Weighted predictions of the proteins in the *Protease* dataset.

The best combination of parameters and feature set for classifying the proteins in the *Protease* dataset was *numberTrees* = 1000, *mtry* = 10, a weight of 20 given to each observation in the in positive class, a random seed of 8716758538734970127 and the following thirty-five features out of the original 105 (S3 Supplementary Information) The positive similarity of the proteins in the *Protease* dataset can be seen in Fig. 7. The distribution of the proteins in the *Protease* dataset likely indicates that there is a strong similarity between the positive and unlabelled proteins, as there are no particularly large peaks in any of the bins more extreme bins (0.0–0.1 and 0.9–1.0). Using a cutoff of 0.5, the RF’s predicted classifications are shown in Table 24.

**Table 24:** Random Forest predicted classifications for Proteases.

Positive Observations				Unlabelled Observations				G Mean
Total	TPs	FNs	Sensitivity	Total	TNs	FPs	Specificity	
59	52	7	0.88	472	419	53	0.89	0.88

As can be seen from Table 25, the distribution of the types of all misclassified proteases closely follows that of the entire *Protease* dataset, rather than the set of positive proteins. However, when only those unlabelled proteases that are most likely to make suitable drug targets are considered, those with a positive similarity ≥0.75, the distribution of the types of the misclassified unlabelled proteins is much closer to that of the positive proteins. For example, although four unlabelled aspartic proteases are misclassified, none of them have a positive similarity ≥0.75. Similarly, although 34% of all misclassified unlabelled proteases are metallo proteases, 50% of misclassified unlabelled proteases with positive similarity ≥0.75 are. The potential drug targets with positive similarity ≥0.75 are also more similar to the positive proteins in terms of their propensity to be membrane bound. While 23% of all misclassified unlabelled proteins are membrane bound, 36% of the unlabelled proteins with positive similarity ≥0.75 are, in close agreement with the 35% of positive proteins that are membrane bound. The small number of misclassified unlabelled proteins with the more confident predictions is likely due to the small number of positive proteins. As the set of positive proteins increases, it is likely that what constitutes similarity to a positive protein will begin to broaden, and more unlabelled proteins will be deemed to be potential drug targets. Similarly, as non-metallo proteases begin to compose more of the set of positive proteins the fraction of the most confident potential drug target predictions that are metallo proteases will likely decrease. All unlabelled proteins in the *Protease* dataset predicted to be positive can be found in S4 Supplementary Information.

**Table 25:** Division of positive and unlabelled proteases by type.

	Aspartic	Cysteine	Metallo	Serine	Threonine
Entire Dataset	31 (6%)	135 (25%)	167 (31%)	161 (30%)	19 (4%)
Unlabelled Proteins	29 (6%)	133 (28%)	131 (28%)	148 (31%)	13 (3%)
Unlabelled Proteins With Positive Similarity >0.5	4 (8%)	9 (17%)	18 (34%)	14 (26%)	5 (9%)
Unlabelled Proteins With Positive Similarity ≥0.75	0 (0%)	2 (14%)	7 (50%)	3 (21%)	2 (14%)
Positive Proteins	2 (3%)	2 (3%)	36 (61%)	13 (22%)	6 (10%)

The distribution of all unlabelled proteins, misclassified unlabelled proteins and all positive proteins by protease type. The 18 unlabelled proteases of an unknown type are not shown.

#### Dataset Homogeneity

In order to further clarify the reasons for the differences in the G means of the RFs, the homogeneity of each dataset was estimated. For each dataset, the pairwise sequence identity between all pairs of proteins in the dataset was calculated using BLAST. The proteins in a pair were considered to be similar if their pairwise sequence identity was at least 20%. This threshold was chosen as it is generally the lowest threshold at which sequence alignments can still be considered reasonable estimates of homology. As the maximum number of similar pairs, excluding identity pairs, for a set of *N* proteins is N2 = N!2!N-2!, the percentage of all possible pairs of two positive proteins, two unlabelled proteins and one positive and one unlabelled protein that are similar could be calculated for each dataset. This percentage indicates the level of similarity between proteins in a given set, with a higher percentage indicating that the proteins in the set are more similar and interconnected. The results of the similarity comparisons can be seen in Table 26.

**Table 26:** Comparison of pairs of proteins with pairwise sequence identity of at least 20%.

Dataset	All Pairs	Pairs of Two Positive Proteins	Pairs of Two Unlabelled Proteins	Pairs of One Unlabelled and One Positive Protein
AllTargets	0.47%	0.92%	0.49%	0.28%
Cancer	1.23%	2.59%	1.29%	0.60%
GPCR	33.41%	35.47%	39.17%	15.38%
GPCR_NO	20.20%	35.47%	16.81%	21.46%
IonChannel	5.27%	9.69%	4.40%	3.66%
Kinase	31.45%	42.44%	30.67%	32.89%
Protease	6.54%	18.81%	6.41%	6.26%

For each dataset, the values indicate the percentage of all possible pairs, pairs consisting of two positive proteins, pairs consisting of two unlabelled proteins and pairs consisting of one positive and one unlabelled protein for which the pairwise sequence identity was at least 20%.

From the results it can be seen that datasets with a large percentage of similar pairs, the *GPCR_NO* and *Kinase* datasets, induce RFs with low G means. The high percentage of similar inter-class pairs is likely to be particularly problematic for a RF’s classifications, as this indicates that the positive and unlabelled proteins are highly similar and likely more difficult to separate and classify well. This problem can best be seen in the differences between the results for the *GPCR* and *GPCR_NO* datasets. While the inter-class similarity is high in the *GPCR* dataset, it is six percentage points less than in the *GPCR_NO* dataset. Additionally, although the 291 non-odorants make up 41% of the unlabelled proteins, they are involved in 57% of the similar inter-class pairs, while the 421 odorants are involved in 43%. The odorants can therefore be seen to be much less similar to the positive proteins than the unlabelled non-odorants, and likely form a highly interconnected cluster separate from a second cluster of non-odorants. The removal of the odorants from the dataset will therefore remove a large source of proteins that were relatively simple to classify correctly, along with highlighting the true similarity between the proteins that have potential to serve as targets (non-odorants). This will result in a substantially lower G mean for the RF trained on the *GPCR_NO* dataset when compared to the one trained on the *GPCR* dataset.

In contrast to the *GPCR_NO* and *Kinase* datasets, the *AllTargets* dataset has very low percentages for all pair types, but still induced a RF with a low G mean. This poor performance indicates that protein datasets can induce poorly performing RFs as a result of being too heterogeneous as well as by being too homogeneous. However, in the case of heterogeneous datasets, it is likely the low level of intra-class similarity that is problematic, as with a low level of intra-class similarity the clustering that the RF relies on to provide accurate classifications is absent. Additionally, highly heterogeneous datasets will cause the individual trees in the forest to show greater variance in their classifications of a given feature subspace, resulting in less confident aggregate predictions.

Although there were no difficulties in obtaining a large G mean for RFs induced from the *Protease* dataset, the large proportion of metallo proteases in the positive proteins, and the high level of similarity between them, could prove problematic. By looking at the similarity between specific subpopulations of the *Protease* dataset (Table 27) it can be seen that the similarities between the proteins in the dataset are largely intra-type, i.e. between two metallo or two non-metallo proteases. Out of the 9231 pairs of proteins in the dataset that are similar, only 185 (2.0%) of the pairs include one metallo and one non-metallo protease. The lack of similarity is particularly striking for the pairs of positive proteins, where only 2 (0.6%) pairs of similar proteins consist of a metallo and non-metallo protease. These results demonstrate that the *Protease* dataset is divided into a minimum of two clusters, one of metallo and one of non-metallo proteases, with the cluster of non-metallo proteases potentially containing further subclusters (such as of serine proteases). While this clustering will prove problematic for the analysis of the important features in the *Protease* dataset, as there is no one cluster of ‘drug target-like proteins’, there is no reason to expect that it would prove to be problematic for the RF classifications, as RFs can easily work with datasets that contain distinct clusters in separate subspaces.

**Table 27:** Similarities between pairs of proteins in the *Protease* dataset.

		Positive		Unlabelled	
		Metallo	Non-metallo	Metallo	Non-metallo
Positive	Metallo	257	2	457	12
	Non-metallo		74	42	1233
Unlabelled	Metallo			583	129
	Non-metallo				6442

Each cell corresponds to one of the possible protein pair combinations. For example, there are 257 pairs consisting of two positive metallo proteases and 129 consisting of an unlabelled metallo protease and an unlabelled non-metallo protease.

## Discussion

### Sequence Identity Comparison

By basing the definition of redundancy on sequence similarity, the proteins in the dataset are being placed in a similarity space, where the distance between any two proteins is related to their pairwise sequence identity. In this space, groups of similar proteins will form clusters, while dissimilar ones will be scattered farther apart. The protein similarity graph captures this information, and simplifies it by only connecting proteins that reach a certain level of similarity. As redundancy removal is achieved by ensuring that no two proteins in the non-redundant dataset share an edge, it conceptually functions by thinning out user-defined clusters of proteins in the similarity space through the replacement of a cluster by a representative subset of its proteins.

There are two situations where this thinning out of clusters is necessary: when you want to generate a representative dataset or when you believe that your dataset is biased. The goal when generating a representative dataset is to cover the same subset of the similarity space covered by the original dataset, maximise coverage, while using as few proteins as possible, minimise ‘redundancy’. Bias in a dataset, in the context of redundancy removal, is taken to mean that the distribution of the proteins in the dataset throughout the similarity space is not the same as the true distribution of the entire population of proteins. Certain similarity subspaces will therefore contain more proteins than they would under the true distribution, and consequently have a disproportionate influence on conclusions drawn from the dataset. Thinning out clusters of proteins in subsections of the similarity space overpopulated due to biases can therefore be used to rebalance the dataset back towards the true distribution.

For our particular application, the generation of a representative set is much less important, due to computational resources not being stretched, than bias removal. However, bias is only a concern when the dataset is a sample that has been drawn from a population of proteins and is being used to draw conclusions about the population. In the case of the *AllTargets* dataset, the population of proteins under consideration is the entire human proteome, while in the case of the *GPCR, IonChannel, Kinase* and *Protease* datasets, the populations are the set of all human GPCRs, ion channels, kinases and proteases respectively. As the dataset being used in all five cases is the same as the population of interest, there is no potential source of selection bias or problems due to generalising to proteins outside the dataset. Unlike the datasets based on protein families, the proteins in the *Cancer* dataset are influenced by past discoveries and historical research preferences, none of which are without bias, and can therefore be seen to be a biased sample of the entire human proteome. However, as no generalisations are being made to proteins outside this biased sample, the conclusions drawn about the proteins in the *Cancer* dataset will not themselves be biased. Therefore, as bias is not a concern for any of the datasets used here, there is no theoretical reason for them to undergo redundancy removal.

Although the similarity based removal of proteins from the datasets is not necessary, the removal of observations from a dataset can potentially improve the quality of an algorithm trained on it. However, in the case of the protein datasets examined here, the best RFs were always induced using the entire dataset, indicating that the removal of proteins with similar sequences does not improve a classifier’s performance. Despite this, there is no indication that measuring similarity between proteins based on their sequences and defining the decision boundary based on the dataset’s features was particularly detrimental. Rather, it was likely the act of removing proteins in general that led to the decrease in performance. Therefore, due to the lack of theoretical need or practical benefit, redundancy removal was avoided when determining the properties of and classifying drug target proteins.

### Target Prediction and Properties

One noticeable trend across the datasets is the lack of effect for features that could be considered to represent interactions between proteins. The primary measures of this were the number of binary PPIs and the number of phosphorylation sites, as phosphorylation sites are indicative of a protein’s involvement in regulatory networks. Despite the biological importance of interactions between proteins, the size of the effect of both the difference in binary PPIs and phosphorylation sites was small for all datasets, with the difference in total phosphorylation sites in the *Cancer* dataset having the largest effect (*PS* = 0.40). Although it is unclear whether interactions between proteins would be expected to be more or less likely to occur in targets, the lack of importance is perhaps surprising given the importance of the regulation of proteins and the interactions between them.

Another set of features that were minimally important across the datasets is the germline variants. Of the four variant types investigated, consequential effects were seen for synonymous coding variants in the *Cancer* dataset (with a very small effect), 3’ and 5’ untranslated region variants in the *Cancer* dataset (both with moderately large effects) and nonsynonymous coding variants in the *AllTargets* and *Cancer* datasets (with very small and moderate effects respectively). The unique pattern of effects for the *Cancer* dataset is predominantly a result of the greater number of variants in the unlabelled proteins, and is most likely due to the characteristics of cancer, rather than the fact that the *Cancer* dataset is composed of proteins implicated in a disease instead of based on protein family membership. Although the number of germline variants is relatively unimportant for current drug targets, were personalised medicine to become commonplace, it is possible that proteins with a larger number of known variants that alter their expression or activity would become more likely to be drug targets, as a greater number of variants would mean that there is more potential for targeting them.

Although the number of PEST motifs and the number of *N*-linked glycosylation sites are both believed to be important in degradation control, their effects do not correlate strongly. As the number of *N*-linked glycosylation sites is greater in the positive proteins for all tested datasets, it would be expected that the positive proteins have fewer PEST motifs, due to them having a longer *in vivo* half-life. However, this is only true for two of the four datasets where the features could be analysed accurately. Even in the datasets where there are fewer PEST motifs in the positive proteins, the effect is always small in both absolute terms and relative to that of the *N*-linked glycosylation sites. These results indicate that there must be some substantial difference in the degradation protection provided by having fewer PEST motifs and more *N*-linked glycosylation sites, or that additional functions of the two are important in helping to determine the differences in their effect sizes.

The clearest difference between positive and unlabelled proteins is in the proportion of non-polar/polar amino acids and in the likelihood of being membrane bound. If the *GPCR_NO* and *IonChannel* datasets are discounted, as they consist solely of transmembrane proteins, the number of transmembrane helices can be seen to have a moderate to large effect for both the *AllTargets* and *Cancer* datasets. When coupled with the size of the effect for the sequence length in the *GPCR_NO* and *IonChannel* datasets, the tendency of proteins to be membrane bound explains the differences in the fraction of non-polar/polar amino acid residues. Being membrane bound is therefore important for, and highly indicative of, a protein being a drug target, and raises the question of whether the results are capturing properties that are truly indicative of being a drug target, or simply of being a membrane bound protein. However, it is believed to be unlikely that the results are simply highlighting properties of membrane bound proteins, since differences in targets and unlabelled proteins arise even when all the proteins are membrane bound [9]. The performances of the RFs would also seem to indicate that there are real differences between the datasets that cannot be explained by the predisposition of targets to being membrane bound, as the difference in transmembrane helices does not correlate strongly with the G mean of the optimised classifier.

### Dataset Homogeneity

The homogeneity of the datasets is of particular importance when attempting to predict potential targets or determine the properties important for the successful targeting of a protein. Of the three datasets that induced poorly performing RFs (*AllTargets, GPCR_NO* and *Kinase*), the *GPCR_NO* and *Kinase* datasets were very homogenous, as seen by the high percentage of pairs of proteins that were similar. Conversely, the *AllTargets* dataset was shown to induce poorly performing RFs due to the heterogeneity of the dataset. The performance of the RFs induced using the *LargeFamilies* and *SmallFamilies* datasets indicate that this is likely due to a combination of the presence of overlapping subpopulations and the difficulty of classifying proteins from smaller families. The differences in the subpopulations likely cause there to be more overlap between clusters of unlabelled and positive proteins, while the proteins from smaller families likely negatively impact the clustering of proteins from larger families. The G mean of the RF trained on the *AllTargets* dataset is also likely to be optimistic for the same reasons that the RF trained on the *GPCR* dataset was. However, in the case of the *AllTargets* dataset there are possibly more subpopulations than just the odorant GPCRs that are overly simple to classify, potentially increasing the favourable bias in the results.

Despite the low G mean of the RFs induced using the *AllTargets, GPCR_NO* and *Kinase* datasets, any features that are determined to have an effect on the likelihood of a protein being a suitable drug target are not invalidated by the homogeneity of the datasets. Rather, features are simply less likely to be found to be important when the positive and unlabelled proteins are excessively similar or dissimilar. Conversely, with the *Protease* dataset the homogeneity of the positive proteins proves to be problematic for the determination of the important features, but not for the capabilities of the RF induced from it. This is because RFs can easily handle datasets with distinct subpopulations, due to their partitioning of the feature space. The cluster of metallo proteases will therefore not influence the RFs performance on the cluster of non-metallo proteases, as the clusters occupy different feature subspaces. The classifications are therefore not unfavourably affected by the large proportion of positive metal proteases. Rather it simply makes the RF better at determining potential metallo protease drug targets than it is at determining potential non-metallo protease targets.

### Random Forests Mitigate the Potential for Overfitting

For most problems, using the same dataset to optimise the parameters, select the optimal feature set and train and evaluate the final classifier would lead to severe overfitting. However, the atypical nature of the problem addressed here lends itself to this approach without risking overfitting. This is because overfitting occurs when a classifier fits limited training observations too closely, and therefore describes the properties of the training set rather than the underlying relationships between features. However, in our case the data available for training is the entire population rather than a sample of it, and there are therefore no observations that can be generalised to. The ideal classifier would therefore be optimised for performance on the training set, as this will optimise the classifier for performance on the entire population.

Despite the lack of need for any generalisation capabilities, we would like to extract potential future drug targets from the unlabelled proteins. This requires the ability to make informed predictions, rather than simply describing the differences between the positive and unlabelled proteins. However, training and evaluating the classifier on the same dataset would in general lead to severely biased predictions. This bias would make unlabelled proteins overly likely to be classified as unlabelled, and vice versa for positive proteins, thereby causing potential drug targets to be missed. We would therefore like to use our entire dataset for both optimising the parameters and training the final classifier, while still being able to use the final classifier to make unbiased predictions about the observations in our dataset.

## Conclusions

Subdividing the entire human proteome in order to create a relatively homogenous subset of proteins is necessary for forming an accurate picture of the features that are important for determining a protein’s drug target likeness. While the heterogeneous *AllTargets* dataset does provide some information about drug targets in general, the effect sizes of the individual features are small and the classifications inaccurate. In contrast, datasets formed from more homogenous subsets generally had features with larger effect sizes, and all induced RFs with greater G means. However, protein datasets can quickly become too homogeneous, negatively impacting the classification capability of a RF trained on them. Care is therefore needed when deciding on a subset of the human proteome to use, as certain subdivisions will produce datasets with vastly different levels of homogeneity. The ideal dataset would have distinct subpopulations (like the *Protease* dataset) or a small but sufficient level of homogeneity (like the *Cancer* and *IonChannel* datasets). This homogeneity does not have to be based on family membership or even disease class, but could come from structural, functional or other properties of proteins instead. Despite the restrictions placed on the datasets by the homogeneity requirements, potential targets can be predicted and properties important for the targeting of proteins determined.

The properties that were most important in differentiating targets from non-targets were found to be the proteins’ hydrophobicities, *in vivo* half-lives, propensity for being membrane bound and the fraction of non-polar amino acids in their sequences. Taken together, the importance of these properties indicates that drug targets are predominantly membrane bound proteins, and therefore non-polar, with long *in vivo* half-lives. However, in the case of the datasets that consist solely of membrane bound proteins, the *GPCR_NO* and *IonChannel* datasets, the targets are predominantly more polar, rather than non-polar, due to the greater proportion of their sequence that resides in the extra and intracellular spaces. Whilst the primary importance of these general properties holds for all datasets, the *Cancer* dataset contained additional properties of secondary importance. These secondary features were predominantly associated with the specific and reliable activity/expression of the proteins (e.g. phosphorylation sites and germline variants), and likely indicate that from amongst all proteins involved in cancer, those with the most specific and reliable activity are preferentially chosen to be antineoplastic targets. As the *Cancer* dataset also showed the most pronounced effect for the importance of the general properties, the range and strength of the importance of the properties in the *Cancer* dataset when compared to the other datasets, along with the high quality of the RF induced using it, likely indicates that subdivisions based on a disease possess the most promise for informative future analysis.

## Supporting Information

S1 Supplementary Information Protein Datasets (XLSX)

S2 Supplementary Information Methods Details (DOCX)

S3 Supplementary Information Features Retained by Random Forests after Using Genetic Algorithm (DOCX)

S4 Supplementary Information Predicted Cancer Target Proteins; Predicted GPCR Target Proteins; Predicted Ion Channel Target Proteins; Predicted Kinase Target Proteins; Predicted Protease Target Proteins (DOCX)
