# Supplementary material for: Properties of Protein Drug Target Classes
Source: PLoS One. 2015 Mar 30;10(3):e0117955. doi: 10.1371/journal.pone.0117955 (PMC4379170; doi:10.1371/journal.pone.0117955)
Supplement: S2 Supplementary Information — (DOCX) [file pone.0117955.s002.docx]

**Properties of Protein Drug Target Classes**

Simon C. Bull & Andrew J. Doig

**SI Document 2 Details on Methods**

**Ensembl Extraction of Information on Alternative Transcripts, Paralogues and Germline Variants**

The first step in extracting the data was to find the set, $E$, of all Ensembl Gene IDs that can be cross-referenced with a representative UniProt human protein accession. The number of protein coding transcripts, $t_{e}$, generated by each gene $e\in E$ was then determined. Once the number of protein coding transcripts associated with each gene was known, the number associated with each representative UniProt human protein accession, $p$, could be calculated. This was done by first finding the set, $E_{p}\subseteq E$, of Ensembl Gene IDs that could be cross-referenced with $p$, and then taking $\max_{e\in E_{p}} t_{e}$ to be the number of protein coding transcripts associated with protein $p$.

The number of paralogues associated with each representative UniProt human protein accession was determined using the Ensembl Perl API. For each Ensembl Gene ID, $e\in E$, the Ensembl Gene IDs, $e_{hom}$, of the genes calculated by Ensembl to be homologous to $e$ were extracted. The set of Ensembl Gene IDs, $e_{par}$, of genes paralogous to $e$ therefore consists of all genes, $h\in e_{hom}$, where the description field in the homology table entry for the homologous relationship between $e$ and $h$ is 'within_species_paralogue’. The number of paralogues of a UniProt accession, $p$, is then calculated by first determining the set of Ensembl Gene IDs, $E_{p}$, that can be cross-referenced with $p$, and then $\#(\bigcup_{e\in E_{p}} e_{par})$, the number of unique paralogues of the genes in $E_{p}$.

The Ensembl Perl API was also used to extract information about the variants associated with each representative UniProt human protein accession. In order to do this, every gene, $e\in E$, was associated with four sets of variants:

- $e_{3}$, the set of variants that occur within the 3’ untranslated region of $e$.
- $e_{5}$, the set of variants that occur within the 5’ untranslated region of $e$.
- $e_{non}$, the set of nonsynonymous coding variants that occur within $e$.
- $e_{syn}$, the set of synonymous coding variants that occur within $e$.

In order to determine these four sets, the set of all variants associated with $e$ is first extracted. This set is then reduced to contain only those variants that have at most one consequence for each transcript coded for by $e$. This cut down set of variants is termed $e_{mut}$. $e_{3}$ is then the set of variants in $e_{mut}$ that occur within the 3’ untranslated region of $e$. $e_{5}$, $e_{non}$ and $e_{syn}$ are formed from $e_{mut}$ analogously. The number of each of the four variant consequences of interest associated with a UniProt accession, $p$, was then calculated by first determining the set of Ensembl Gene IDs, $E_{p}$, that can be cross-referenced with $p$. The number of 3’ untranslated region variants associated with $p$ is then $\sum_{e\in E_{p}} \#e_{3}$, the number of 5’ untranslated region variants $\sum_{e\in E_{p}} \#e_{5}$, the number of nonsynonomous coding variants $\sum_{e\in E_{p}} \#e_{non}$ and the number of synonymous coding variants $\sum_{e\in E_{p}} \#e_{syn}$.

**Extraction of UniProt Accession Numbers**

In order to determine the representative UniProt accessions of the approved targets in the DrugBank database, the XML file of the database was first parsed to determine the set, $D$, of approved small molecule drugs. A drug, $d$, was only included in $D$ if the **type** attribute of its <drug> element was "small molecule" and the data associated with one of the <group>child elements of its <groups> child element was ‘approved’. For each drug $d\in D$, the DrugBank target IDs, $T_{d}$, of the proteins that it targets were determined by extracting the **partner** attribute of each <target> child element of $d$’s entry in the XML file. The set of DrugBank target IDs of every target of an approved small molecule is then $T=\bigcup_{d\in D} T_{d}$. Following this, the file of external database cross-references (http://www.drugbank.ca/system/downloads/current/drug_links.csv.zip accessed July 8th 2013) was parsed to determine the set, $U$, of UniProt accessions of the targets in $T$. The accessions in $U$ were then converted to a set of representative accessions, $U_{DB}$, using the non-representative to representative accession mapping.

The TTD database was parsed to extract the UniProt accessions of the targets of approved drugs (those targets with an identifier beginning with ‘TTDS’) recorded in the database. The accessions were subsequently converted to a set, $U_{TTD}$, of representative accessions using the non-representative to representative accession mapping. The accessions in $U_{TDD}-U_{DB}$, i.e. those accessions solely implicated as targets by the TTD, were then further analysed to ensure that the proteins they identify are the target of an approved small molecule drug.

**Feature Selection Using a Genetic Algorithm**

A CHC-GA combines a conservative selection strategy, preserving the best individuals after every generation, with a crossover operator that is both disruptive and produces offspring that are maximally different to their parents. Given a set of features, $F$, and the number of individuals to generate in each generation, $P$, the algorithm first sets the $threshold$ parameter to $\#F/4$. The $threshold$ parameter controls how dissimilar two parents must be before they can be combined to produce offspring via crossover. Parents that are too similar are thus prevented from mating.

Following the initialisation of the $threshold$ parameter, the initial population $P_{0}$ is created. Each individual in $P_{0}$ is initialised to be a set of $\#F/2$ features. The distribution of the features throughout $P_{0}$ is controlled in such a way that for every pair of features, $i,j\in F$, the number of individuals in $P_{0}$ that contain $i$ is at most one more than the number that contains $j$. This ensures that, provided the size of the population is large enough, no feature’s influence is curtailed due to the vagaries of the initialisation process. Following the initialisation of $P_{0}$, the fitness of each individual, $p_{i}\in P_{0}$, is calculated by growing a RF from the features in $F-p_{i}$. The G mean is then calculated from the OOB predictions of the RF, with larger values for the G mean corresponding to fitter individuals. The RF is grown using the previously determined optimal parameters.

After initialising $P_{0}$ and calculating the fitness of each individual in it, the generational portion of the GA can begin. The first step in each generation, $g$, is to select the pairs of parents, $M_{g}$, that will undergo crossover in order to produce a set of offspring, $O_{g}$. This is done by randomly selecting $P/2$ pairs of individuals, with replacement, from $P_{g}$. In order to undergo crossover, the Hamming distance between the parents in a given pair, $\left( p_{i},p_{j} \right)\in M_{g}$, must be greater than the $threshold$. As individuals in $P_{g}$ are sets of features, the Hamming distance between any two individuals is equal to the number of features in their symmetric difference, i.e. $\#(p_{i}\triangle p_{j})$. Provided that the parent pairing is permitted to undergo crossover, half uniform crossover is used to produce two offspring by swapping a random subset of half the features that differ between the parents, i.e. half the features in $p_{i}\triangle p_{j}$. Assuming that the set of features to be swapped is $s$, the two offspring, $o_{i}$ and $o_{j}$, are created such that $o_{i}=p_{i}\triangle s$ and $o_{j}=p_{j}\triangle s$. If no offspring are created through crossover, then the $threshold$ is decremented by one. This ensures that future pairs of parents do not have to be as dissimilar as the pairs in the current generation. Once the $threshold$ reaches 0 the run stops as the population is deemed to have converged.

After $O_{g}$ has been produced, the fitness of each individual in it is calculated in the same manner used for the individuals in $P_{0}$. The population for the next generation, $P_{g+1}$, is then determined from $O_{g}$ and $P_{g}$ by setting $P_{g+1}$ to be the fittest individuals from $P_{g}\cup O_{g}$, such that $\#(P_{g})=\#(P_{g+1})$. It is possible, especially when a run nears convergence, that the members of $O_{g}$ will all be less fit than the members of $P_{g}$, causing $P_{g}=P_{g+1}$. If this occurs repeatedly the run can get stuck in a loop, whereby the $threshold$ does not decrease because the individuals in $P_{g}$ are not too similar, but the offspring are not fit enough to make it into $P_{g+1}$. In order to prevent this, a check is inserted that causes the $threshold$ to be decremented by one if a set number of generations pass without the population changing.

## Pairwise Sequence Identity Comparison

The pairwise sequence identity between all pairs of sequences in the human proteome (extracted from UniProt) was determined using PSI-BLAST version 2.2.25, from the NCBI BLAST+ package, with the default scoring matrix. Each protein was BLASTed sequentially against every other protein in order to determine all $N^{2}$ possible sequence identities. The arguments used for the BLASTing were:

- -evalue 1
- -inclusion_ethresh 0.0001
- -num_iterations 3
- -gap_trigger 18
- -num_descriptions 10000
- -num_alignments 10000
- -dbsize 0
- -outfmt "7 qseqid sseqid pident length evalue"

For each of the five categories, non-redundant datasets were created using the Leaf algorithm {Bull, 2013 #1932} with pairwise sequence identity thresholds of 20%, 30%, 40%, 50%, 60%, 70%, 80%, 90% and 100%. A non-redundant dataset generated using a threshold of 100% contains all proteins in the category, i.e. no proteins are considered to be redundant. For each combination of pairwise sequence identity and category, redundancy was removed from the set of positive proteins and the set of unlabelled proteins in the category separately. The non-redundant positive and unlabelled proteins were then combined to make the final non-redundant dataset. This method allows for both members of a similar pair to be kept, if one is in the positive set and one in the unlabelled set.

Each of the nine non-redundant datasets generated for a category underwent the same evaluation process. For a dataset, $d$, this process was:

1. Optimise the positive class weighting, with $mtry=10$.
2. Perform feature selection using the CHC-GA algorithm with $mtry=10$, $numberTrees=1000$ and the positive class weighting found in step 1. The CHC-GA was allowed to converge once, and the feature subset/random seed pair that induced the RF with the greatest G mean was taken to be the optimal combination.
3. Train a RF using the optimal positive class weighting, feature subset and random seed, along with $mtry=10$ and $numberTrees=1000$.
4. Using the RF generated in step 3, predict the class of each protein in $d$ using the forest’s OOB predictions, and the class of the redundant proteins in the category using the entire forest.
5. Use the predictions generated in step 4 to calculate the G mean of the forest.

For each category, the threshold that produced the dataset that led to the largest G mean in step 5 was deemed to be the optimal threshold to use. The optimal threshold is therefore the one that enables the induction of a RF that forms the best predictions of all proteins in the category.
