# Supplementary material for: Properties of Protein Drug Target Classes
Source: PLoS One. 2015 Mar 30;10(3):e0117955. doi: 10.1371/journal.pone.0117955 (PMC4379170; doi:10.1371/journal.pone.0117955)
Supplement: S3 Supplementary Information — (DOCX) [file pone.0117955.s003.docx]

**Properties of Protein Drug Target Classes**

Simon C. Bull & Andrew J. Doig

**SI Document 3 Features Retained by Random Forests after Using Genetic Algorithm**

**All Proteins**

- Amino acid compositions
  - The proportion of cysteine, glycine, leucine, serine, tyrosine and aromatic residues.
- Simple sequence properties
  - The hydrophobicity, isoelectric point, number of low complexity regions, presence of a signal peptide and sequence length.
- Posttranslational modifications
  - The number of *N*-linked glycosylation, *O*-linked glycosylation and phosphotyrosine sites.
- Secondary structure
  - The fraction of residues predicted to participate in $\beta$-strands, buried $\alpha$-helices and exposed $\alpha$-helices.
  - The number of transmembrane $\alpha$-helices.
- Germline variants
  - The number of 3’ untranslated region variants
- Inter-protein relationships
  - The number of binary PPIs.
- Developmental stage expression:
  - The neonate expression level.
- Body site expression
  - The bladder, brain, connective tissue, ear, embryonic tissue, esophaus, eye, heart, larynx, liver, lung, salivary gland, skin, spleen, testis, tonsil, umbilical cord and uterus expression levels.
  - The number of body sites that the protein is expressed in.

**Cancer Proteins**

Amino acid compositions

- - The proportion of alanine, arginine, asparagine, cysteine, glycine, histidine, methionine, proline, serine, tryptophan and aromatic residues.
- Simple sequence properties
  - The presence of a signal peptide.
- Posttranslational modifications
  - The number of *N*-linked glycosylation, phosphothreonine and phosphotyrosine sites.
- Secondary structure
  - The fraction of residues predicted to participate in $\beta$-strands and buried $\alpha$-helices.
  - The number of transmembrane $\alpha$-helices
- Germline variants:
  - The number of 3’ untranslated variants.
- Inter-protein relationships
  - The number of paralogues.
- Developmental stage expression
  - The blastocyst, neonate and juvenile expression levels.
- Body site expression
  - The adrenal gland, brain, cervix, ear, embryonic tissue, heart, lymph node, prostate, skin, stomach, testis and uterus expression levels

**GPCRs**

Amino acid compositions

- - The proportion of asparagine, glycine, histidine, isoleucine, serine, tryptophan, valine, aliphatic and non-polar residues.
- Simple sequence properties
  - The number of PEST motifs and the presence of a signal peptide.
- Posttranslational modifications
  - The number of *N*-linked glycosylation, *O*-linked glycosylation and phosphothreonine sites.
- Secondary structure
  - The fraction of residues predicted to participate in $\beta$-strands, buried $\alpha$-helices and exposed $\alpha$-helices.
- Germline variants
  - The number of 5’ untranslated variants.
- Inter-protein relationships
  - The number of alternative transcripts and paralogues.
- Developmental stage expression
  - The embryoid body and neonate expression levels.
- Body site expression
  - The bone, bone marrow, brain, mammary gland, mouth, salivary gland, testis, thymus, thyroid, trachea, umbilical cord, uterus and vascular expression levels.
  - The number of body sites that the protein is expressed in.

**GPCR_NO**

- Amino acid compositions
  - The proportion of glutamine, histidine, proline, serine, negatively charged and polar residues.
- Simple sequence properties
  - The sequence length and presence of a signal peptide.
- Posttranslational modifications
  - The number of *O*-linked glycosylation, phosphothreonine and phosphotyrosine sites.
- Secondary structure
  - The fraction of residues predicted to participate in $\beta$-strands and buried $\alpha$-helices.
  - The number of transmembrane $\alpha$-helices.
- Germline variants
  - The number of 3’ untranslated variants.
- Inter-protein relationships
  - The number of paralogues and binary PPIs.
- Developmental stage expression
  - The embryoid body, blastocyst, fetus and infant expression levels.
- Body site expression
  - The adrenal gland, bladder, blood, bone, brain, connective tissue, ear, embryonic tissue, eye, intestine, kidney, liver, lung, nerve, ovary, placenta, prostate, spleen, stomach, testis, thymus, thyroid, tonsil, umbilical cord and uterus expression levels.
  - The number of body sites that the protein is expressed in.

**Ion Channels**

Amino acid compositions

- - The proportion of alanine, cysteine, glycine, lysine, phenylalanine, tryptophan and tiny residues.
- Simple sequence properties
  - The number of PEST motifs, presence of a signal peptide and sequence length.
- Posttranslational modifications
  - The number of *O*-linked glycosylation and phosphoserine sites.
- Secondary structure
  - The fraction of residues predicted to participate in $\beta$-strands.
- Germline variants
  - The number of 5’ untranslated, nonsynonymous coding and synonymous coding variants
- Inter-protein relationship
  - The number of alternative transcripts.
- Developmental stage expression
  - The blastocyst and juvenile expression levels.
- Body site expression
  - The ascites, bone, esophagus, intestine, kidney, liver, lung, lymph, lymph node, mouth, muscle, nerve, ovary, pancreas, pharynx, prostate, thymus, thyroid, umbilical cord, uterus and vascular expression levels.

**Kinases**

Amino acid compositions

- - The proportion of asparagine, aspartic acid, cysteine, glycine, lysine, methionine, serine, tryptophan, aliphatic, charged, negatively charged and non-polar residues.
- Simple sequence properties
  - The number of PEST motifs.
- Posttranslational modifications
  - The number of *N*-linked glycosylation, *O*-linked glycosylation, phosphothreonine and phosphotyrosine sites.
- Inter-protein relationships
  - The number of binary PPIs.
- Developmental stage expression
  - The embryoid body, fetus, neonate and juvenile expression levels.
- Body site expression
  - The bone, cervix, ear, liver, lymph node, parathyroid, salivary gland, thymus, umbilical cord and uterus expression levels.

**Proteases**

Amino acid compositions

- - The proportion of arginine, cysteine, isoleucine, glycine, proline, tryptophan and aromatic residues.
- Simple sequence properties
  - The number of PEST motifs.
- Posttranslational modifications
  - The number of *O*-linked glycosylation, *N*-linked glycosylation, phosphothreonine and phosphotyrosine sites.
- Secondary structure
  - The fraction of residues predicted to participate in buried $\alpha$-helices.
- Germline variants
  - The number of 3’ and 5’ untranslated variants.
- Inter-protein relationship
  - The number of paralogues.
- Developmental stage expression
  - The embryoid body, neonate and infant expression levels.
- Body site expression
  - The adipose tissue, blood, bone, cervix, connective tissue, ear, embryonic tissue, mouth, nerve, placenta, prostate, salivary gland, thymus, trachea, umbilical cord and uterus expression levels.
