# Supplementary material for: Properties of Protein Drug Target Classes
Source: PLoS One. 2015 Mar 30;10(3):e0117955. doi: 10.1371/journal.pone.0117955 (PMC4379170; doi:10.1371/journal.pone.0117955)
Supplement: S4 Supplementary Information — (DOCX) [file pone.0117955.s004.docx]

**Properties of Protein Drug Target Classes**

Simon C. Bull & Andrew J. Doig

**SI Document 4 Predicted Target Proteins**

Cancer Proteins

| Accession | Positive Similarity | Accession | Positive Similarity |
| --- | --- | --- | --- |
| Q13635 | 1.00 | P60568 | 0.87 |
| Q9HC73 | 0.99 | P31994 | 0.85 |
| O95436 | 0.99 | O15393 | 0.83 |
| P08922 | 0.99 | Q93063 | 0.83 |
| Q9UKU0 | 0.99 | P20151 | 0.82 |
| Q16288 | 0.99 | O60674 | 0.82 |
| Q86VZ1 | 0.99 | P05091 | 0.82 |
| P25106 | 0.98 | Q99643 | 0.82 |
| Q96JT2 | 0.98 | P24864 | 0.80 |
| Q9UM73 | 0.97 | Q16549 | 0.79 |
| Q9BQ51 | 0.96 | Q8WVQ1 | 0.79 |
| P53675 | 0.95 | Q06136 | 0.78 |
| Q6UXM1 | 0.95 | P10415 | 0.78 |
| Q9NZQ7 | 0.95 | P02786 | 0.75 |
| P40259 | 0.94 | Q9UKJ5 | 0.74 |
| P42702 | 0.94 | P43405 | 0.74 |
| P48735 | 0.93 | Q9Y5W5 | 0.74 |
| O95573 | 0.93 | P18074 | 0.74 |
| P51654 | 0.92 | Q8NG68 | 0.73 |
| P42336 | 0.91 | Q16394 | 0.69 |
| Q9HBE5 | 0.91 | Q9NPI8 | 0.67 |
| P36894 | 0.90 | P55287 | 0.66 |
| P11912 | 0.90 | O14521 | 0.66 |
| Q9Y693 | 0.90 | Q9ULV8 | 0.64 |
| Q008S8 | 0.89 | P12830 | 0.63 |
| O75874 | 0.89 | Q9HB96 | 0.59 |
| Q9H2T7 | 0.88 | Q01860 | 0.59 |
| Q8NFG4 | 0.88 | Q92989 | 0.58 |
| Q96PJ5 | 0.88 | Q99983 | 0.58 |
| P49589 | 0.88 | O15360 | 0.57 |
| Q00597 | 0.87 | Q02223 | 0.52 |
| P14222 | 0.87 | P35125 | 0.51 |

GPCRs

| Accession | Positive Similarity | Accession | Positive Similarity |
| --- | --- | --- | --- |
| O15303 | 0.93 | P21730 | 0.72 |
| O43613 | 0.92 | Q9UHM6 | 0.70 |
| O60883 | 0.91 | Q9BPV8 | 0.70 |
| Q99705 | 0.89 | Q15391 | 0.69 |
| Q9NYM4 | 0.89 | Q9Y5X5 | 0.69 |
| Q8IZP9 | 0.88 | Q9UBY5 | 0.68 |
| O43614 | 0.87 | P51684 | 0.68 |
| P49190 | 0.86 | Q99680 | 0.68 |
| O95838 | 0.85 | P32241 | 0.65 |
| Q86Y34 | 0.85 | P34998 | 0.65 |
| P41146 | 0.82 | Q16538 | 0.64 |
| P30989 | 0.81 | Q15761 | 0.64 |
| Q15760 | 0.80 | O15354 | 0.64 |
| Q9BZJ8 | 0.80 | Q9NPB9 | 0.63 |
| Q96P65 | 0.79 | P51686 | 0.63 |
| Q9NSD7 | 0.79 | Q9H461 | 0.61 |
| Q8IZ08 | 0.79 | Q99677 | 0.60 |
| Q8IZF7 | 0.77 | Q8IZF4 | 0.60 |
| P32247 | 0.77 | Q9BZJ7 | 0.60 |
| P48546 | 0.76 | Q9UP38 | 0.58 |
| P25090 | 0.76 | Q96K78 | 0.58 |
| P29371 | 0.75 | Q7RTX1 | 0.57 |
| P41586 | 0.75 | Q9H1Y3 | 0.57 |
| P41587 | 0.74 | P21453 | 0.53 |
| Q9GZQ4 | 0.72 | O95977 | 0.53 |

Ion Channels

| Accession | Positive Similarity |
| --- | --- |
| Q7Z3S7 | 0.96 |
| Q15878 | 0.96 |
| Q03721 | 0.84 |
| Q7Z442 | 0.84 |
| Q401N2 | 0.83 |
| Q99712 | 0.81 |
| Q01118 | 0.81 |
| A5X5Y0 | 0.79 |
| O95264 | 0.76 |
| Q9NPI9 | 0.76 |
| Q9NTG1 | 0.74 |
| Q9UHC3 | 0.74 |
| O00168 | 0.70 |
| Q9UQD0 | 0.69 |
| Q7Z443 | 0.69 |
| Q8TD43 | 0.65 |
| P63252 | 0.63 |
| O60928 | 0.63 |
| Q8IZF0 | 0.61 |
| Q9Y210 | 0.52 |
| Q70Z44 | 0.50 |

Kinases

| Accession | Positive Similarity | Accession | Positive Similarity | Accession | Positive Similarity |
| --- | --- | --- | --- | --- | --- |
| Q16288 | 0.99 | P36897 | 0.79 | Q8N4C8 | 0.66 |
| P09769 | 0.98 | P54756 | 0.79 | P78368 | 0.65 |
| P41743 | 0.97 | P51812 | 0.79 | Q05513 | 0.65 |
| Q06187 | 0.97 | Q16539 | 0.79 | P05129 | 0.65 |
| Q05397 | 0.96 | P08237 | 0.78 | Q6XUX3 | 0.65 |
| Q15375 | 0.95 | P42356 | 0.78 | Q8TAS1 | 0.64 |
| P41240 | 0.95 | Q9UF33 | 0.78 | Q00535 | 0.64 |
| P54764 | 0.94 | P19367 | 0.76 | P51451 | 0.62 |
| Q9Y4K4 | 0.94 | O00329 | 0.76 | Q01974 | 0.62 |
| P21860 | 0.93 | Q15139 | 0.76 | Q13418 | 0.62 |
| P54760 | 0.92 | P08922 | 0.76 | Q13163 | 0.62 |
| Q15303 | 0.92 | P21709 | 0.76 | P34925 | 0.62 |
| P29320 | 0.91 | P35590 | 0.75 | O43353 | 0.62 |
| P42680 | 0.91 | P17858 | 0.74 | Q9NVE7 | 0.62 |
| P54753 | 0.91 | P42681 | 0.74 | Q01973 | 0.61 |
| P29323 | 0.91 | Q13705 | 0.73 | P15531 | 0.60 |
| Q8TD19 | 0.90 | P22392 | 0.73 | Q9NY57 | 0.60 |
| P43405 | 0.89 | O43252 | 0.73 | Q9HC98 | 0.59 |
| Q06418 | 0.88 | Q9BZL6 | 0.73 | Q9UEW8 | 0.59 |
| Q15746 | 0.88 | Q16644 | 0.73 | P46734 | 0.59 |
| P50750 | 0.88 | P68400 | 0.73 | Q15418 | 0.59 |
| Q12866 | 0.87 | P43403 | 0.72 | Q16877 | 0.59 |
| Q13308 | 0.87 | Q00534 | 0.71 | P53350 | 0.58 |
| P30530 | 0.87 | Q9UHD2 | 0.70 | Q9HBH9 | 0.58 |
| P29597 | 0.86 | P06493 | 0.70 | P45984 | 0.58 |
| P45983 | 0.86 | Q9H479 | 0.69 | Q9BUB5 | 0.57 |
| O75716 | 0.84 | P27037 | 0.69 | O14976 | 0.54 |
| P14616 | 0.84 | P51813 | 0.69 | O14936 | 0.54 |
| Q14680 | 0.83 | Q96GD4 | 0.68 | P34947 | 0.53 |
| P24723 | 0.83 | Q8IVH8 | 0.67 | Q8TDX7 | 0.53 |
| Q01813 | 0.83 | P11802 | 0.67 | Q8WZ42 | 0.53 |
| O95340 | 0.82 | Q13546 | 0.67 | P52429 | 0.53 |
| Q13557 | 0.82 | P31751 | 0.67 | Q38SD2 | 0.53 |
| P00558 | 0.81 | Q04759 | 0.67 | Q16875 | 0.52 |
| P53671 | 0.80 | Q9UBF8 | 0.67 | P78527 | 0.51 |
| P36894 | 0.80 | P50613 | 0.66 |  |  |

Proteases

| Accession | Positive Similarity | Accession | Positive Similarity |
| --- | --- | --- | --- |
| Q9UKU6 | 0.93 | P48052 | 0.65 |
| P15169 | 0.90 | Q9Y6M0 | 0.65 |
| Q12884 | 0.84 | Q9NVE5 | 0.65 |
| P25787 | 0.81 | Q8N6M6 | 0.65 |
| Q96T52 | 0.81 | P52948 | 0.64 |
| Q9UQQ1 | 0.80 | P43234 | 0.64 |
| Q9UI42 | 0.79 | P25788 | 0.63 |
| Q9HBA9 | 0.77 | Q9UHL4 | 0.63 |
| Q9NY33 | 0.77 | O14672 | 0.62 |
| P17655 | 0.76 | P49720 | 0.61 |
| P42892 | 0.75 | Q8IUX7 | 0.61 |
| Q53GS9 | 0.75 | Q9Y6W3 | 0.61 |
| P06681 | 0.75 | Q96FV2 | 0.60 |
| P28072 | 0.75 | P29122 | 0.57 |
| P12955 | 0.74 | Q8NBH2 | 0.57 |
| O95084 | 0.73 | P55786 | 0.57 |
| P61009 | 0.73 | Q14623 | 0.57 |
| Q8TB40 | 0.69 | P0DJD8 | 0.57 |
| Q86TI2 | 0.69 | Q9Y3Q0 | 0.56 |
| Q9H3G5 | 0.68 | Q9UJW2 | 0.56 |
| P05156 | 0.68 | Q9Y5B9 | 0.55 |
| P00736 | 0.68 | O96009 | 0.55 |
| Q9Y5Z0 | 0.67 | Q7Z2K6 | 0.54 |
| O43895 | 0.67 | P55210 | 0.53 |
| Q2TV78 | 0.67 | Q53RT3 | 0.50 |
| Q99436 | 0.66 | P07384 | 0.50 |
| Q9UQ90 | 0.65 |  |  |
